# Coral-associated nitrogen fixation rates and diazotrophic diversity on a nutrient-replete equatorial reef

**DOI:** 10.1038/s41396-021-01054-1

**Published:** 2021-07-22

**Authors:** Molly A. Moynihan, Nathalie F. Goodkin, Kyle M. Morgan, Phyllis Y. Y. Kho, Adriana Lopes dos Santos, Federico M. Lauro, David M. Baker, Patrick Martin

**Affiliations:** 1 Earth Observatory of Singapore, Interdisciplinary Graduate School, Nanyang Technological University, Singapore, Singapore; 2 Asian School of the Environment, Nanyang Technological University, Singapore, Singapore; 3 Earth Observatory of Singapore, Nanyang Technological University, Singapore, Singapore; 4 American Museum of Natural History, New York, NY USA; 5 Singapore Centre for Environmental Life Sciences Engineering (SCELSE), Nanyang Technological University, Singapore, Singapore; 6 Division for Ecology and Biodiversity, School of Biological Sciences, University of Hong Kong, Hong Kong, PR China; 7 The Swire Institute of Marine Science, University of Hong Kong, Hong Kong, PR China

**Keywords:** Microbial ecology, Biogeochemistry, Stable isotope analysis

## Abstract

The role of diazotrophs in coral physiology and reef biogeochemistry remains poorly understood, in part because N_2_ fixation rates and diazotrophic community composition have only been jointly analyzed in the tissue of one tropical coral species. We performed field-based ^15^N_2_ tracer incubations during nutrient-replete conditions to measure diazotroph-derived nitrogen (DDN) assimilation into three species of scleractinian coral (*Pocillopora acuta*, *Goniopora columna*, *Platygyra sinensis*). Using multi-marker metabarcoding (16S rRNA, *nifH*, 18S rRNA), we analyzed DNA- and RNA-based communities in coral tissue and skeleton. Despite low N_2_ fixation rates, DDN assimilation supplied up to 6% of the holobiont’s N demand. Active coral-associated diazotrophs were chiefly Cluster I (aerobes or facultative anaerobes), suggesting that oxygen may control coral-associated diazotrophy. Highest N_2_ fixation rates were observed in the endolithic community (0.20 µg N cm^−2^ per day). While the diazotrophic community was similar between the tissue and skeleton, RNA:DNA ratios indicate potential differences in relative diazotrophic activity between these compartments. In *Pocillopora*, DDN was found in endolithic, host, and symbiont compartments, while diazotrophic *nifH* sequences were only observed in the endolithic layer, suggesting a possible DDN exchange between the endolithic community and the overlying coral tissue. Our findings demonstrate that coral-associated diazotrophy is significant, even in nutrient-rich waters, and suggest that endolithic microbes are major contributors to coral nitrogen cycling on reefs.

## Introduction

Nitrogen (N) is considered the major limiting nutrient on coral reefs, with dissolved inorganic nitrogen (DIN) concentrations typically <1 µM [1]. Corals nevertheless create highly productive and diverse ecosystems [2], which is largely attributed to efficient carbon and nitrogen recycling with their endosymbiotic algae (Symbiodiniaceae). Corals also harbor a diverse microbial community, comprised of Bacteria, Archaea, and single-celled Eukarya [3]. Although direct observations of these microorganisms are sparse, they can be major sources of nutrients for corals [4, 5]. Among coral-associated microorganisms, dinitrogen (N_2_) fixing Bacteria and Archaea, known as diazotrophs, are believed to play an important role in coral physiology and reef biogeochemistry [6]. Diazotrophs are a source of new nitrogen, and coral-associated diazotrophs can contribute up to 18% of gross benthic N_2_ fixation in shallow fore-reef environments [7]. Diazotrophs have been found in bulk analyses of the coral microbial community [8], and assimilation of diazotroph-derived nitrogen (DDN) has been documented within the coral mucus, host tissue, algal symbionts, and skeleton [8–11].

Despite several rate-based observations of N_2_ fixation in corals, the significance, variability, and fate of coral-associated N_2_ fixation remain poorly understood. Several studies observed that the majority of DDN was acquired by Symbiodiniaceae [8, 12–14] and could meet up to 11% of their N demand [15]. Yet, others found little to no transfer of fixed nitrogen to Symbiodiniaceae, but reported that DDN was lost to the water column [9, 16] (n.b. results from [16] may have been underestimated [17] by use of the bubble method [18]). In comparing species, one study found that autotrophic corals (*Pocillopora verrucosa*, *Stylophora pistillata*) rely more on DDN than heterotrophic corals (Fungiidae) [10]. Other studies have highlighted the role of heterotrophy in DDN assimilation [19], and significant rates of diazotroph ingestion and DDN assimilation have been observed in *Stylophora pistillata* [14, 20], particularly during bleaching [20]. However, environmental conditions have an uncertain role in coral-associated diazotrophy. While N_2_ fixation rates appear to increase with temperature [21, 22], DDN assimilation in coral has been observed to both increase [19, 20] and decrease [23] in bleached corals. During bleaching, DDN is hypothesized to either alleviate bleaching stress and provide an alternative source of N, compensating for a reduced translocation of N from Symbiodiniaceae to the host [9, 19, 20], or to actually disrupt the coral symbiosis and trigger bleaching by changing the nutrient supply ratio to Symbiodiniaceae [24, 25]. Moreover, N_2_ fixation rates and diazotrophic community composition have only been jointly analyzed in the tissue of one tropical coral species from nutrient-poor waters [8]. Given the distinct differences in coral morphology and microbial community composition between coral species, as well as the wide range of environmental conditions under which coral reefs form, our understanding of this potentially important microbial process is limited.

The majority of coral-associated N_2_ fixation measurements have been made in subtropical, oligotrophic waters (e.g., Red Sea) using aquaria, with DIN concentrations ranging from 0.17 to 1.4 µM and dissolved inorganic phosphorus (DIP) concentrations from 0.02 to 0.3 µM, when reported (Table S1). N_2_ fixation was thought to occur primarily in DIN-depleted waters [26, 27], with coral-associated diazotrophy increasing in response to seasonal oligotrophic conditions [15]. However, recent findings suggest that N_2_ fixation is likely underestimated in DIN-replete coastal waters [28–30] and that in some cases, nitrate may even enhance planktonic [31, 32] and coral-associated [33] diazotrophy. In addition, the ratio of DIN to DIP, which has rarely been reported in coral-diazotrophy studies (Table S1), may control N_2_ fixation more than the DIN concentration alone [34]. As coral reefs form within a range of nutrient conditions, it is critical to constrain rates of N_2_ fixation for a variety of reef settings, including reefs impacted by human activities and urbanization, as well as in understudied regions, such as Southeast Asia [6, 35, 36]. Moreover, all previous measurements of coral-associated N_2_ fixation have been performed in aquaria (Table S1). As coral microbial communities can change drastically within a day of aquarium captivity [37, 38], in situ studies are a critical next step toward fully understanding coral-associated N_2_ fixation.

Here, we performed 24-h incubations using reef-acclimated fragments of three hard coral species on two reefs in Singapore. Studies were conducted during the southwest monsoon, when nutrient concentrations are seasonally elevated. We measured rates of DDN assimilation into four separate coral compartments (host tissue, algal symbionts, released mucus, skeleton), and constrained the contribution of coral-associated N_2_ fixation for both the holobiont and reef nitrogen budgets. Additionally, we analyzed the DNA- and RNA-based microbial community composition in both the coral tissue and the skeleton, using a multi-marker metabarcoding approach. As RNA has a shorter lifespan than DNA, comparison of RNA and DNA can be used to gain insight into the relative activity of taxonomic groups and biogeochemical processes that scale with cell production [39–43]. Using RNA:DNA ratios, we examine the relative activity of key taxa between coral species and compartments, in relation to isotopic N_2_ fixation rate measurements.

## Materials and methods

### Sample collection and incubation

Corals were sampled at Pulau Hantu (1°13′38.6 N, 103°44′48.1 E) and Kusu Island (1°13′31.6 N 103°51′37.5 E), Singapore (Fig. S1) at 3–5 m depth. Two species were used at each site (Kusu: *Pocillopora acuta* and *Platygyra sinensis*; Hantu: *Goniopora columna* and *Platygyra sinensis*) and are referred to throughout the text by their genus. Species were selected based on trophic position and relative abundance at each reef. Benthic cover data were compiled from recent studies [44, 45]. Per species per site, four colonies were fragmented underwater into pieces with ~30 cm^2^ of living tissue surface area. Two fragments per colony (*n* = 32) were attached to an underwater rig at ~4 m depth using cable ties and allowed to acclimate for 1 week. Of these fragment pairs, one fragment from each colony was incubated in enriched water and the other in unenriched water. At the time of fragmentation, additional samples were collected from each colony, stored on ice, and then stored at −20 °C (*n* = 16). These fragments were used to determine baseline isotopic signatures to detect any effect of fragmentation and acclimatization on individual coral colonies.

Incubations were performed with dissolved ^15^N_2_ gas [17]. Prior to each experiment, ~80 l of surface water were collected from each reef and filtered sequentially through GF/F (Whatman-1825-047) and 0.2 µm cellulose acetate filters (Whatman-7001-0004). This filtered water was used in coral incubations, filter-sterilized controls, and to prepare the ^15^N_2_ label. The ^15^N_2_ label (Cambridge Isotopes) was prepared following Klawonn et al. [46] (see Supplementary Information), achieving a final enrichment >90% ^15^N_2_.

Four ^15^N_2_ enriched and four unenriched incubations were performed per species per site (32 total coral incubations). Prior to incubation, fragments were attached to clean PVC holders with new cable ties and rinsed briefly with filter-sterilized seawater. As enrichment controls, filtered-sterilized seawater was enriched and incubated in triplicate. Using a Niskin bottle, seawater was collected on the day of the experiment at 5 m depth, the approximate depth of the reef, and was enriched and incubated in triplicate to measure water column N_2_ fixation. All incubations were performed in 1 l glass jars with Teflon-lined lids (Wheaton-Z263486). Jars were sealed, headspace-free, while submerged in water, and 100 ml of pre-dissolved ^15^N_2_ tracer (>90% ^15^N_2_, described above) were injected into enriched incubations via septa to achieve a final enrichment of 8.5–11.5% ^15^N_2_. A separate septum and needle were used as an outflow while injecting the labeled water. The enrichment in each incubation jar was measured directly, as described below. Prior to deployment, dissolved oxygen was measured in each jar (Unisens-OXY1-SMA).

Jars were attached to crates and incubated on the reef for 24 h (~13:00–13:00) (Fig. S2). After retrieval, collection and filtration of both seawater and coral samples were performed immediately aboard the boat. In each jar, dissolved oxygen was measured and seawater was sampled for atom% ^15^N_2_, using He-flushed Exetainers (Labco-938W) with 10 µl 50% wt/vol ZnCl_2_ per ml of sample as a preservative. The remaining seawater in each jar was then filtered onto pre-combusted, pre-weighed GF/F filters for isotopic analysis of coral mucus released into the water during the incubation [19, 47], referred to as “released mucus”. With a pore size of 0.7 µM, GF/F filters capture mucus aggregates, but may miss smaller, non-aggregated cells [48]. Each coral fragment was photographed for photogrammetric surface area measurements (Autodesk-ReCap Photo), and then split into subsamples using a sterilized chisel. Approximately 1/4 of each coral was flash-frozen in a dry shipper and stored at −80 °C for molecular analysis, and the rest was transported on ice and stored at −20 °C for isotopic analysis.

A Niskin bottle was used to collect additional seawater samples (5 m depth). In total, 1 l was filtered onto a 0.22 µm filter to sample the water column microbial community (Sterivex-SVGP01050), and 1 l was filtered onto pre-combusted, pre-weighed GF/F filters to measure particulate *δ*^15^N and *δ*^13^C. Samples for dissolved inorganic nutrients were filtered through 0.22 µm polyethersulfone syringe filters (Pall-4612) into acid-washed 15 ml tubes. Seawater samples were flash-frozen in a dry shipper and stored at −80 °C (microbial) and −20 °C (nutrients) until analysis.

### Isotope analysis

Isotopic analysis was performed on four coral compartments: host tissue, algal symbionts, released mucus, and skeleton. Coral tissue for isotope analysis was thoroughly removed with ultrapure water (Elga, 18.2 MΩ cm^−1^) using an airbrush (GSI Creos, Procon) and homogenized with a rotor-stator tissue homogenizer (BioSpec-985370-14). The homogenate was centrifuged at 700 rcf, separating the algal pellet (symbiont) from the supernatant (host). The supernatant was spun at 3000 rcf to further purify the coral host tissue and pellet any remaining symbiont fraction. After the initial separation, the symbiont pellet was rinsed twice with ultrapure water at 1500 rcf and once at 2000 rcf. Tissue was freeze-dried, weighed in silver capsules (Sercon-SC0035), and acidified with 6 N HCl prior to analysis (Thermo Scientific-TS-24308). After tissue removal, remaining tissueless skeletons were dried at 60 °C for 48 h. The endolithic layer (Fig. S2b) was removed from tissueless samples using a Dremel tool and a diamond disc. Care was taken to remove the upper portion of the skeleton, particularly in *Platygyra* samples, by “skimming” this uppermost portion off the skeleton. Endolithic samples were cryopulverized (BioSpec-59014N), dried at 60 °C for 24 h, and weighed into tin capsules (Sercon-SC0148). Filters with released coral mucus and seawater particulate matter were dried at 60 °C for 24 h, weighed, and folded into tin capsules.

Particulate isotope samples were analyzed using a Eurovector Elemental Analyzer and Nu Instruments Perspective Isotope Ratio Mass Spectrometer at the University of Hong Kong. Instrumental error was <0.16‰ and determined using an in-house acetanilide standard (iACET), certified by Indiana University. *δ*^15^N and *δ*^13^C values were determined using Eqs. (1) and (2):

1 $$\delta ^{15}{{{\rm{N}}}} (\textperthousand) = \left[\frac{{R_{{{{\mathrm{sample}}}}}}}{{R_{{{{\mathrm{air}}}}}}} - 1\right] \times 1000$$

2 $$\delta ^{13}{{{\rm{C}}}}(\textperthousand) = \left[\frac{{R_{{{{\rm{sample}}}}}}}{{R_{{{{\rm{PDB}}}}}}} - 1\right] \times 1000$$

where *R*_sample_ = (^15^N /^14^N) and *R*_air_ = 0.003676 for N isotopes, and *R*_sample_ = (^13^C /^12^C) and *R*_PDB_ = 0.011180 for C isotopes. *δ*^15^N was converted to atom% (*A*) using Eq. (3) [18]:

3 $$A = {{{\rm{atom}}}}{{{\mathrm{\% }}}}{\,}^{15}{{{\rm{N}}}} = 100 \;\times \frac{{{\,}^{15}{{{\rm{N}}}}}}{{{\,}^{15}{{{\rm{N}}}} + \,{\,}^{14}{{{\rm{N}}}}}} = 100\; \times \frac{{R_{{{\rm{{air}}}}} \times (\delta {\,}^{15}{{{\rm{N}}}} \times 0.001 + 1)}}{{1 + R_{{{\rm{{air}}}}} \times (\delta {\,}^{15}{{{\rm{N}}}} \times 0.001 + 1)}}$$

with R 4.0.2 [49], compartment- and species-specific paired *t*-tests were used to test for any effect of fragmentation and acclimatization on the isotopic signatures of corals. Isotopic results between species and sites were compared with unpaired *t*-tests. Shapiro–Wilk tests were used to check for normality.

Atom% of dissolved ^15^N_2_ was measured at the University of California Davis using a GasBench and Precon gas concentration system interfaced to an isotope-ratio mass spectrometer. Dissolved ^15^N_2_ measurements were performed for each enriched incubation chamber and in non-enriched seawater.

### Calculation of N_2_ fixation rate

To determine N_2_ fixation rates, particulate atom% ^15^N from paired enriched (*A*_e_) and control (*A*_c_) replicate samples were subtracted for each fraction (i.e., host tissue, algal symbionts, released mucus, skeleton, and seawater). Detectable N_2_ fixation was defined as when the difference between (*A*_e_) and (*A*_c_) was greater than 3x the standard deviation of repeated *A*_c_ measurements, following [19, 47, 48]. Rates below the detection limit were assumed to be zero when calculating average rates, resulting in more conservative estimates (see Supplementary Information). DDN_assmilation_ was determined following Eq. (4) [18]:

4 $${{{\rm{DDN}}}}_{{{\rm{{assimilation}}}}} = \frac{1}{{{{{{\mathrm{{\Delta}}}} }}t}}\frac{{A_{{{\rm{e}}}} - A_{{{\rm{c}}}}}}{{A_{15{{{\rm{N}}}}2} - A_{{{\rm{o}}}}}} \times \left[ {{{\rm{{PN}}}}} \right]$$

where (*A*_e_−*A*_c_) is the particulate difference in atom%, *A*_15N2_ is the atom%^15^N of each labeled incubation, *A*_o_ is the atom% ^15^N of unlabeled seawater, and [PN] is the particulate nitrogen concentration. [PN] was determined from the %N of the enriched sample. Rates were standardized by dry weight (DW), surface area, and/or volume, according to the sample type (see Supplementary Information refs. [4–6]).

### Water column chemistry

Dissolved nutrients were measured using a SEAL AA3 autoanalyzer following standard methods [50]. The detection limits were 0.05, 0.03, 0.12, and 0.10 µM for NO_*x*_, PO^4^_3−_, NH^+^_4_, and Si(OH)_4_, respectively. DIN is taken as [NO_*x*_] + [NH_4_^+^]. DIP is [PO^4^_3_^−^]. Temperature, salinity, and chlorophyll-a at the incubation site were measured using a Valeport FastCTD profiler with chlorophyll fluorometer. Photosynthetically active radiation (PAR) was measured during a concurrent experiment [51], ~1 m shallower than the incubation experiment.

### Nucleic acid extraction, PCR, sequencing, and bioinformatics

For nucleic acid extraction, coral tissue (host and symbiont) was removed with an autoclaved, 0.22 µm-filtered 1x PBS, 10 µM EDTA solution using an airbrush and homogenized with a rotor-stator tissue homogenizer. The coral skeletons were cryopulverized. Tissue and skeletal samples were aliquoted for DNA and RNA extractions. Detailed protocols of sample preparation, nucleic acid extractions, and PCR conditions are reported in the Supplementary Information.

PCR was performed using the following primer sets: 16S rRNA (V6-V8 hypervariable region), B969F 5′- ACGCGHNRAACCTTACC-3′ and BA1406 5′-ACGGGCRGTGWGTRCAA-3′ [52] (cf. [53–55]); nitrogenase iron protein (*nifH*), IGK3 5′-GCIWTHTAYGGIAARGGIGGIATHGGIAA-3′ and DVV 5′-ATIGCRAAICCICCRCAIACIACRTC-3′ [56]; 18S rRNA (V4 hypervariable region) (coral samples) UNonMet-F 5′-GTGCCAGCAGCCGCG-3′ and UNonMet-R 5′-TTTAAGTTTCAGCCTTGCG-3′ [57], followed by V4-18S-For 5′-CCAGCASCYGCGGTAATTCC-3′ and V4-18S-Rev 5′-ACTTTCGTTCTTGATYRATGA-3′ [58]; and 18S rRNA (seawater samples) V4-18S-For and V4-18S-Rev. Samples were purified, barcoded, and sequenced by the GeT-PlaGe platform of GenoToul (INRA Auzeville, France) using an Illumina MiSeq platform (2 × 250 bp).

Sequence data were processed in R 4.0.2 [49] using DADA2 [59, 60], as described in the Supplementary Information. For the 16S rRNA and 18S rRNA datasets, the SILVA 16S rRNA database (v138) [61, 62] and the PR^2^ database (v4.12) [63] (https://pr2-database.org/) were used as training sets. For *nifH*, a new database was created in DADA2 format by modifying, updating, and reformatting the *nifH* June 2017 ARB database from Heller et al. [64]. Files, scripts, and documentation used in creating the *nifH* DADA2 database can be found at https://github.com/moyn413/nifHdada2 [65]. Sequences were submitted to the NCBI Sequence Read Archive (BioProject: PRJNA678423). Analyses were performed using the R Phyloseq [66] and DESEQ2 packages [67], Geneious Prime 2019.2.3 (https://www.geneious.com), and the Interactive Tree of Life [68]. Files and scripts are available at https://github.com/moyn413/Singapore-coral-microbes.

For 16S rRNA RNA:DNA ratios (16S RNA:DNA), normalized replicates were merged and ratios were determined for each ASV that appeared in both the RNA and DNA samples. Average RNA:DNA ratios of ASVs were determined within taxonomic orders for each coral species and compartment. Due to low replicate numbers in paired *nifH* RNA and DNA samples, coral species and compartment were not analyzed separately for *nifH* RNA:DNA ratios. As RNA:DNA ratios are known to have high variability within and between taxonomic groups [40, 42], ratios should be interpreted with caution.

## Results

### Site characterization

DIN ranged from 2.80 to 3.48 µM, DIP from 0.21 to 0.23 µM, and chlorophyll-a from 0.53 to 0.97 µg l^−1^ (Table S2). This exceeds eutrophication thresholds for coral reefs (DIN: 1 µM; phosphate: 0.1 µM; chlorophyll-a: 0.4–0.5 µg l^−1^ [1, 69]). The N:P ratio was 15.3 (Kusu) and 13.8 (Hantu), slightly below the Redfield ratio (16:1). Salinity (29.6 ± 0.5 psu) and temperature (29.4 ± 0.1 °C) were consistent at both sites throughout the study period (Table S2). The PAR daily light integral was 12.08 mol photons m^−2^ per day (Hantu) and 19.02 mol photons m^−2^ per day (Kusu) on each incubation day [51]. Hantu had significantly lower live hard coral cover (39.1 ± 4.8%) than Kusu (52.8 ± 6.1%) [44] (Table S3). *Goniopora* dominated coral cover at Hantu (12.3%), while Kusu reef had greater hard coral diversity and evenness, with no one species accounting for more than 4% of coral cover [45] (Table S3).

### Baseline isotope ratios and physiology

**Fig. 1:**
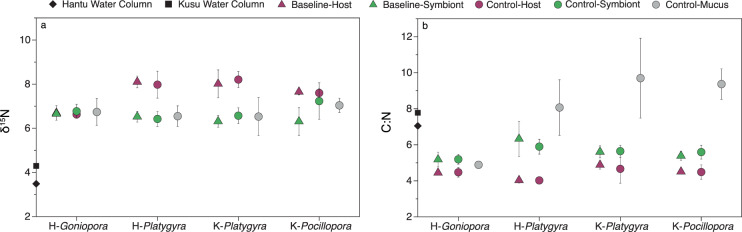
Comparison of δ^15^N values and C:N ratios from Baseline and Control samples. Baseline and Control **a** *δ*^15^N and **b** C:N of coral host tissue, algal symbionts, and released mucus are plotted alongside  water column particulate organic matter *δ*^15^N and C:N from incubation dates. Released coral mucus samples are from Control incubations only.

The *δ*^15^N and *δ*^13^C values of coral fragments collected directly from the reef (Baseline) did not differ from corresponding fragments in non-enriched control incubations (Control) for each species and site (Fig. 1a and Table S4) (paired *t*-tests, *p* > 0.05), indicating that fragmentation and incubation did not affect the isotopic composition. Host tissue *δ*^15^N from Platygyra was ~1.6‰ more enriched than algal symbionts, whereas in *Pocillopora* and *Goniopora*, host tissue was 0.9‰ and –0.05‰ more enriched than the algal symbionts, respectively (Fig. 1a). *δ*^13^C differed significantly between all corals (unpaired *t*-tests, *p* < 0.05), and *Platygyra* from Hantu and Kusu were more ^13^C enriched (–14.3 ± 1.0‰; –15.6 ± 0.6‰) than *Goniopora* and *Pocillopora* (–17.5 ± 0.5‰; –19.8 ± 0.4‰) (Fig. S3). Based on *δ*^15^N and *δ*^13^C results, *Platygyra* was relatively more heterotrophic while *Pocillopora* and *Goniopora* were more autotrophic [70]. These trophic positions were stable over a 2-year period (2017–2019) at the same sites, with host tissue *δ*^15^N greater than symbiont *δ*^15^N by an average of 1.77 ± 0.97‰ (*Platygyra*-Hantu), –0.38 ± 0.34‰ (*Goniopora*-Hantu), 1.57 ± 0.78‰ *Platygyra*-Kusu), and 0.09 ± 0.90‰ (*Pocillopora*-Kusu) (Moynihan, unpublished data). The C:N ratio of host tissue and algal symbionts was lower than that of the released mucus in both Kusu species (unpaired *t*-tests, *p* < 0.05) and in *Platygyra* from Hantu (unpaired *t*-test, *p* = 0.06) (Fig. 1b). In contrast, the C:N ratio of *Goniopora* tissue (host and symbiont) did not differ from that of the released mucus and was lower than all other samples (unpaired *t*-tests, *p* < 0.05).

With the exception of *Pocillopora*, oxygen concentrations decreased in most coral incubations, with average net change in [O_2_] of –3.5 mg l^−1^ (Hantu-*Goniopora*), –1.8 mg l^−1^ (Hantu-*Platygyra*), –0.9 mg l^−1^ (Kusu-*Platygyra*), and +0.2 mg l^−1^ (Kusu-*Pocillopora*). One ^15^N_2_ labeled *Goniopora* replicate experienced anoxia (0.22 mg O_2_ l^−1^) (Fig. S4).

### N_2_ fixation

#### Coral N_2_ fixation

**Fig. 2:**
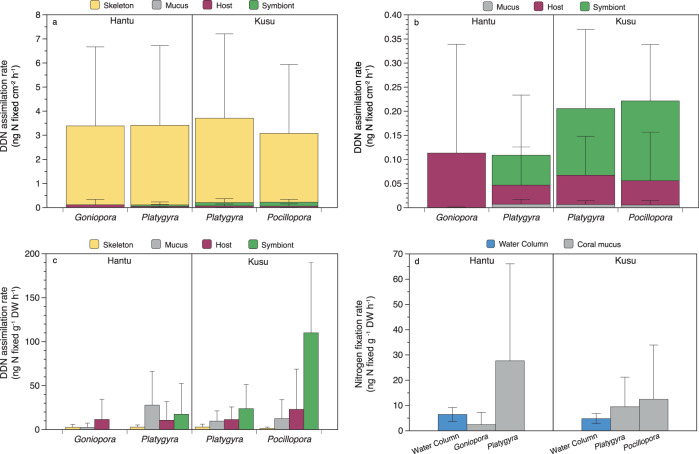
Compartment-specific assimilation rates of diazotroph-derived nitrogen (DDN). DDN assimilation rates  in the skeletal endolithic layer, host tissue, algal symbionts, and released mucus were standardized by **a** surface area (ng N cm^−2^ h^−1^), with the skeleton excluded in **b**, and **c** by dry weight (DW) (ng N g^−1^ DW h^−1^). **d** Nitrogen fixation rates in released coral mucus and water column particulate organic matter, standardized by dry weight (ng N g^−1^ DW h^−1^). Error bars represent the standard deviation in all panels.

High heterogeneity was observed between replicate samples, with 1–3 out of 4 replicates per species having detectable DDN in each compartment (Table S5). When standardized by surface area, DDN assimilation rates were highest in the endolithic skeletal compartment (Fig. 2a), ranging from 0 to 8.3 ng N cm^−2^ h^−1^. Symbiont DDN assimilation ranged from 0–0.32 ng N cm^−2^ h^−1^, and accounted for the majority of non-skeletal assimilation in *Platygyra* and *Pocillopora*. Host assimilation ranged from 0 to 0.45 ng N cm^−2^ h^−1^, and accounted for the majority of non-skeletal assimilation in *Goniopora*. Released mucus assimilation accounted for the least DDN of all compartments in all species (range: 0–0.02 ng N cm^−2^ h^−1^) (Fig. 2b). Normalizing DDN assimilation rates to DW changed relative assimilation rates in the compartments owing to differences in densities between the coral tissue and skeleton. Although not quantified here or in similar studies [9, 23], it would be more accurate to standardize endolithic rates by the skeletal organic content for comparison with other compartments. Normalized to DW, DDN assimilation was greater in the symbiont, released mucus, and host tissue compartments compared to the skeleton (Fig. 2c). N_2_ fixation rates in the released coral mucus were variable but similar to those of the water column (Fig. 2d).

To assess the relevance of these rates for coral nutrition, we estimated the holobiont N demand based on the total N content of bulk tissue and endolithic layer, assuming a tissue growth rate of 0.2% of DW per day, as reported for coral tissue in low-light environments [71, 72]. Endolithic growth rates are likely lower than tissue growth rates [73], which means that our calculation likely overestimated endolithic N demand. Considering the maximum DDN assimilation rates from all four compartments (host tissue, algal symbionts, released mucus, skeleton), N_2_ fixation could account for up to 2.9–6.4% of the total holobiont N demand (Table 1). When skeletal N_2_ fixation is excluded, the remaining N_2_ fixation accounted for <3% of N demand. If all fixed nitrogen in the skeleton were translocated to the tissue above, this could meet up to 5.6–32.5% of the tissue N demand.

**Table 1:** N_2_ fixation as a function of daily nitrogen (N) demand.

Coral growth N budget	Hantu		Kusu	
	*Goniopora*	*Platygyra*	*Platygyra*	*Pocillopora*
Tissue N content (mg N cm^−2^)	1.85 ± 0.50	0.62 ± 0.27	0.49 ± 0.12	0.26 ± 0.09
Skeletal N content (mg N cm^−2^)	1.76 ± 1.12	1.54 ± 0.54	2.17 ± 1.20	1.06 ± 0.19
Total N_2_ fixation/Total N demand	2.89%	3.72%	3.51%	6.45%
Tissue N_2_ fixation/Tissue N demand	0.29%	0.83%	1.25%	2.22%
Skeletal N_2_ fixation/Skeletal N demand	5.62%	4.89%	4.01%	7.49%
Total N_2_ fixation/Tissue N demand	5.56%	12.96%	19.15%	32.54%

Tissue and skeletal N demand were both assumed to be 0.2% dry weight per day, as reported for coral tissue in low-light environments [71, 72]. Skeletal content includes both organic and inorganic nitrogen. Maximum N_2_ fixation rates from the skeleton and tissue (host, symbiont, mucus) were used in calculations.

#### Water column and reef N_2_ fixation budget

Water column N_2_ fixation rates were normalized to dry mass, for comparison with mucus-associated fixation rates, and ranged from 2.5 to 9.3 ng N g^−1^ h^−1^ (0.04–0.1 ng N l^−1^ h^−1^) (Fig. 2d). Assuming a mucus-associated N_2_ fixation rate of 0.005 ng N cm^−2^ h^−1^, as typical for our sites and species, released coral mucus DDN could add 0.06–0.08 nmol l^−1^ of nitrogen to the water column per day, given the estimated areal coral cover (Table S6). Water column N_2_ fixation contributed 0.14 and 0.12 nmol l^−1^ of nitrogen per day at Hantu and Kusu, respectively. While these rates are low, mucus-associated N_2_ fixation at Hantu and Kusu represented 44–67% of the water column N_2_ fixation (Table S6).

### Coral microbial community

**Fig. 3:**
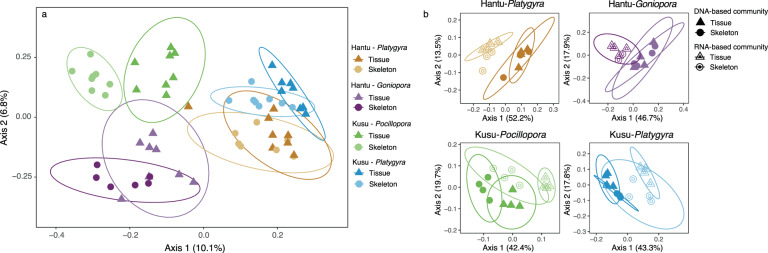
Principal coordinates analysis (PCoA) of coral 16S rRNA microbial community composition. **a** PCoA of combined DNA-based and RNA-based communities, and **b** unifrac PCoA of 16S rRNA DNA-based and RNA-based community samples. Samples are colored by site and species, and shapes indicate tissue (△) or skeletal (○) samples.

In the 16S rRNA community, all alpha diversity indices were greater in the coral tissue (host and symbiont) than the endolithic skeleton compartment (Table S7). The DNA-based communities of Hantu-*Goniopora* and Kusu-*Platygyra* tissues were significantly more diverse than that of their skeletons, as well as the RNA-based community of *Platygyra* (Kusu) (unpaired *t*-tests, *p* < 0.05). Coral species influenced the 16S rRNA microbial community composition more than sample type (i.e., tissue or skeleton) (Fig. 3a). However, within each species, significant differences were observed between tissue and skeletal communities (PERMANOVA-Adonis) (Kusu-*Pocillopora*, *R*^2^ = 0.41, *p* = 0.001; Kusu-*Platygyra*, *R*^2^ = 0.30, *p* = 0.004; Hantu-*Platygyra*, *R*^2^ = 0.31, *p* = 0.004; Hantu-*Goniopora*, *R*^2^ = 0.38, *p* = 0.01) (Fig. 3b). Moreover, the DNA-based 16S rRNA community differed from the RNA-based 16S rRNA community in all species (Kusu-*Pocillopora*, *R*^2^ = 0.13, *p* = 0.02; Kusu-*Platygyra*, *R*^2^ = 0.10, *p* = 0.02; Hantu-*Platygyra*, *R*^2^ = 0.16, *p* = 0.001; Hantu-*Goniopora*, *R*^2^ = 0.27, *p* = 0.001) (Fig. 3b).

In DNA-based 16S rRNA communities, Alphaproteobacteria (Rhodobacterales, Rhizobiales) and Bacteroidia (Cytophagales, Flavobacteriales) were abundant, with the common coral-associated genus *Ruegeria* [74] accounting for 51% of Rhodobacterales amplicon sequence variants (ASVs) (Figs. S5 and S6). Within the phylum Cyanobacteria, 78% of ASVs belonged to the order Synechococcales. In RNA-based 16S rRNA communities, the relative percentages of Gammaproteobacteria, Deltaproteobacteria, Bacteroidia, and Cyanobacteria were higher, while the contribution of Alphaproteobacteria was lower (Figs. S5 and S6). Within the Alphaproteobacteria RNA-based community, Rhodobacterales, Rhodospirillales, Rhizobiales, and Kiloniellales were the most abundant orders, with *Ruegeria* accounting for only 18% of Rhodobacterales. Synechococcales and Pleurocapsales, and Oscillatoriales were the most abundant cyanobacterial ASVs in RNA samples, including the known N_2_-fixing filamentous taxa *Hormoscilla* and *Trichodesium*, as well as non-filamentous Pseudanabaenaceae and Xenococcaceae. Plastid 16S rRNA ASVs from the endolithic algae *Ostreobium* were highly abundant in *Pocillopora* skeletal samples (Fig. S7).

Using a UNonMet and 18S rRNA nested primer approach [57], the 18S community was primarily composed of Symbiodiniaceae; only 81 of 1216 taxa were metazoan. Symbiodiniaceae Clade D/E was the dominant clade in *Pocillopora* and *Goniopora*, and Clade C was dominant in *Platygyra* from both sites (Fig. S8). For the 18S rRNA region sequenced, Clade D/E cannot be distinguished. Communities differed between species (PERMANOVA-Adonis, *R*^2^ = 0.66, *p* = 0.001) (Fig. S9) and the average alpha diversity was greater in the skeleton than tissue in RNA-based community samples (unpaired *t*-test, *p* = 0.01). Aside from Symbiodiniaceae, the most abundant microeukaryotes were Bacillariophyta (diatoms), Apicomplexa (parasitic alveolates), Spirotrichea (ciliates), and Ascomycota (fungi) (Fig. S10).

In the seawater community, Bacteria and Archaea were primarily Synechococcales, SAR11, SAR86, Flavobacteriales, Rhodobacterales, and Nitrosopumilales (Fig. S11). Plastid 16S and 18S rRNA sequences were dominated by the diatom genera *Skeletonema* and *Chaetoceros*, green algae of the genera *Osterococcus*, *Bathycoccus*, and *Micromonas*, and dinoflagellates of the classes Syndiniophyceae and Dinophyceae (Fig. S12).

### Diazotrophic *nifH* community

**Fig. 4:**
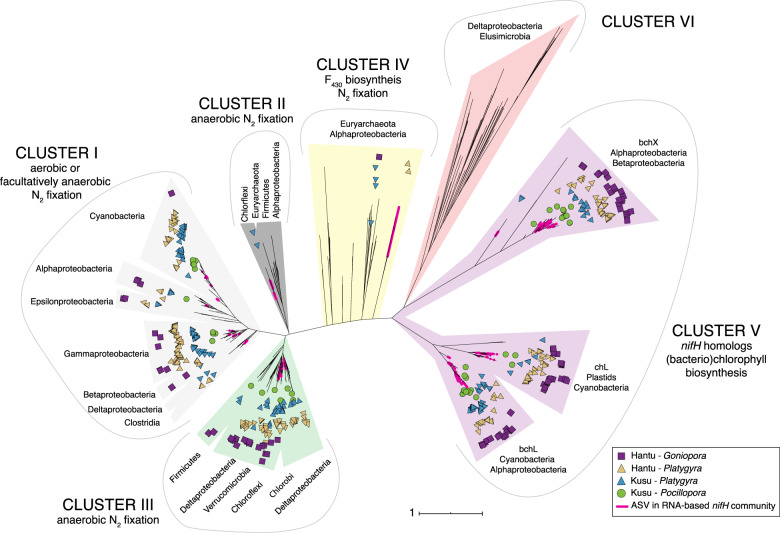
Maximum-likelihood *nifH* phylogeny with ASVs from coral DNA and RNA samples, made using reference sequences from [75, 76], and [64] (see Supplementary Information for detail). ASVs from RNA-based community samples are pink, and ASVs from DNA samples are symbolized by () Hantu-*Goniopora*, () Hantu-*Platygyra*, () Kusu-*Platygyra*, and () Kusu-*Pocillopora*. Clusters I–III are known to fix nitrogen.

**Fig. 5:**
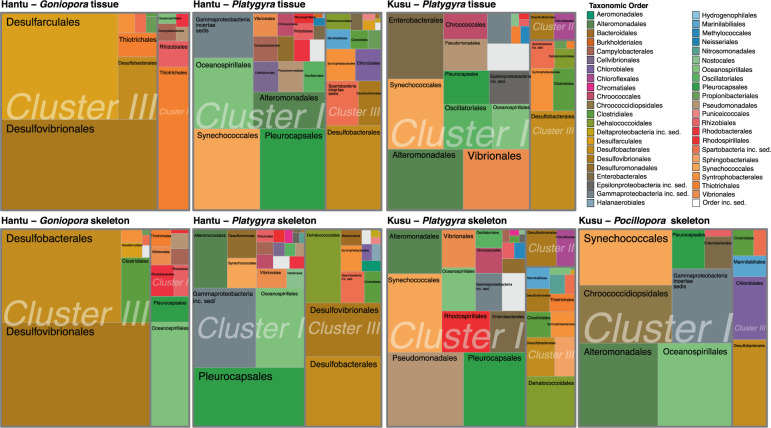
Treemap of Clusters I–III ASVs of the *nifH* DNA-based community for each site, species, and site. Rectangles within each treemap are proportional to the relative abundance of each taxa. Taxa are nested within larger rectangles (separated by gray lines), representing the relative abundance of each cluster. Note that no Clusters I–III ASVs from *Pocillopora* tissue samples were identified.

*nifH* ASVs were classified into six clusters [75]. Most ASVs belonged to Cluster V, which contains *nifH* homologs involved in (bacterio)chlorophyll synthesis (*bchL*, *bchX*, *chlL*) [76, 77] (Figs. 4 and S13). Within Cluster V, ASVs were closely associated with chloroplasts (notably, *Ostreobium* chloroplasts) or with Alphaproteobacteria (Rhizobiales, Rhodobacterales). Within Clusters I–III, which are known to fix nitrogen, Deltaproteobacteria, Gammaproteobacteria, and Cyanobacteria accounted for 86.5% of ASVs in the DNA-based community (Fig. 5).

The relative abundance of Clusters I–III ASVs differed between species (PERMANOVA-Adonis, *R*^2^ = 0.20, *p* = 0.001), but within each species, no difference was found between DNA-based tissue and skeletal communities (PERMANOVA-Adonis) (*p* > 0.05). *Goniopora*’s diazotrophic community was primarily composed of Cluster III sulfate-reducing Deltaproteobacteria and had the lowest abundance of cyanobacterial *nifH* ASVs, which were absent from the tissue (Fig. 5). In *Platygyra*, Cyanobacteria, Gammaproteobacteria, Deltaproteobacteria, and Chloroflexi were the most abundant diazotrophs (Fig. 5), and Chloroflexi were more abundant in the skeleton relative to the tissue at both sites. The relative abundance of ASVs from Clusters I–III in *Platygyra* was 21.0% at Hantu and 14.2% at Kusu. In *Pocillopora*, no *nifH* ASVs from Clusters I–III were found in the tissue, while Clusters I–III represented <1% of the total *nifH* community in the skeleton (predominately Gammaproteobacteria and Cyanobacteria).

Due to practical and technical limitations, the RNA-based *nifH* community was only analyzed in 1/3 of coral samples (4× *Platygyra* and 3× *Goniopora*) (Fig. S14b). In the RNA-based community, Cluster I (aerobes or faculative anaerobes) had the highest relative abundance (Fig. S14b), accounting for 81.1% of Clusters I–III ASVs, including Synechococcales, Pleurocapsales, Oscillatoriales, Chloroflexales, Oceanospirillales, Alteromonadales, Vibrionales, Chromatiales, Desulfuromonadales, Rhizobiales, and Rhodospirillales.

In the water column, only three *nifH* ASVs known to fix nitrogen were found with raw abundances >10, and all were most closely related to Gammaproteobacteria from Cluster I. Although Cluster III ASVs were present in seawater samples, their raw abundances were all below the abundance threshold. The majority of *nifH* sequences from seawater were from Cluster V, and related to Rhodobacterales (*Hyphomonadaceae*), Rhizobiales (*Bradyrhizobiaceae*), and red algal plastids.

### Coral tissue and skeletal relative microbial activity

**Fig. 6:**
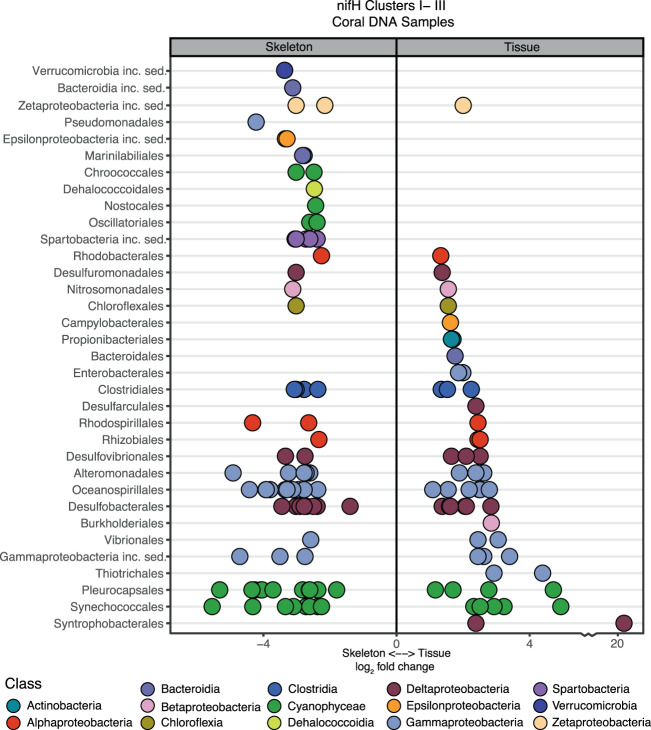
Differential gene abundance analysis (DESEQ2) [113] of *nifH* ASVs from Clusters I to III, between the tissue and skeleton of coral samples. As *Pocillopora* had no *nifH* Clusters I–III ASVs in tissue sequences, it was excluded from this analysis. Results are plotted along a log_2_ scale, with an axis break to include an ASV of the order Syntrophobacterales.

Taxonomic groups in the *nifH* community that differed between tissue and skeleton (DESEQ2 analysis) (Figs. 5 and 6) were analyzed for relative activity using RNA:DNA ratios (Fig. S15). As the 16S rRNA-derived dataset had a higher number of RNA replicates than the *nifH* RNA-based dataset, skeleton and tissue comparisons were made with 16S rRNA data. Overall, patterns evident in 16S rRNA RNA:DNA ratios (16S RNA:DNA) between the skeleton and tissue were similar to patterns observed in the differential abundance (DESEQ2) analysis, suggesting both a change in abundance and relative activity of certain taxa within coral species and compartments. Cyanobacterial 16S RNA:DNA ratios indicated relative activity in both the tissue and skeleton (Fig. S15), particularly in both *Platygyra* species, where an 16S RNA:DNA ratio of 568.7 was observed for an ASV closely related to MBIC10086 of the family Pseudanabaenaceae. Synechococcales, which include N_2_-fixing taxa, were present in skeletal and tissue RNA and DNA samples from all species, except in *Pocillopora* tissue. For *Goniopora*, 16S RNA:DNA ratios of Synechococcales were <1, which is consistent with the low relative abundance of cyanobacteria in the *nifH* community of *Goniopora*. Greater relative activity and variability were observed in cyanobacterial ASVs in the skeleton (16S RNA:DNA = 20.5 ± 95.6 SD, *n* = 35) relative to the tissue (16S RNA:DNA = 4.7 ± 10 SD, *n* = 55). Additional taxonomic orders identified in the *nifH* cyanobacterial community also had high relative activity (16S RNA:DNA > 5), including Pleurocapsales, Chroococcales, and Oscillatoriales (Figs. 5 and S15). Chloroflexi were relatively active in both the tissue and skeleton, and the majority of ASVs had greater relative activity in the skeleton (8.2 ± 31.3 SD, *n* = 34) in comparison with the tissue (3.6 ± 3.9 SD, *n* = 12) (Fig. S15). Sulfate-reducing Deltaproteobacteria had similar 16S RNA:DNA relative activity in both the tissue (5.9 ± 9.6 SD, *n* = 18) and skeleton (3.8 ± 3.6 SD, *n* = 20). Among selected diazotrophic taxonomic groups, the majority of ASVs belonged to the class Alphaproteobacteria or Gammaproteobacteria, with an increased number of relatively active (16S RNA:DNA > 1) selected Gammaproteobacterial ASVs in the tissue (*n* = 207) relative to the skeleton (*n* = 61), whereas the majority (68%) of selected Alphaproteobacteria ASVs had an 16S RNA:DNA < 1. Within Alphaproteobacteria, Rhodospirillales relative activity was highest (Fig. S15), with tissue 16S RNA:DNA exceeding skeletal 16S RNA:DNA, with ratios up to 43.8 (*Goniopora* tissue). Rhizobiales 16S RNA:DNA ratios were relatively low, with the exception of *Goniopora* where N_2_ fixing Pseudoxanthobacter had a ratio of up to 24.2 in the skeleton, whereas relatively active tissue-associated Rhizobiales were associated with a family of methanotrophs (Methyloligellaceae). Within Gammaproteobacteria, orders Oceanospirillales and Alteromonadales were abundant in the *nifH* community and also had elevated 16S RNA:DNA ratios in the tissue and skeleton of all samples. Thiotrichales, which were abundant in the DNA-based *nifH* community of *Goniopora*, were also detected in *Goniopora* 16S RNA:DNA ratios.

RNA:DNA ratios of Clusters I–III *nifH* were analyzed for those samples where *nifH* RNA sequences were measured (*n* = 7). Similarly to the 16S RNA:DNA, *nifH* RNA:DNA suggested relative activity of ASVs of Synechococcales, Gammaproteobacteria (Oceanospirillales, Alteromonadales), and Chloroflexi (Fig. S16).

## Discussion

### Compartment-specific DDN assimilation

**Fig. 7:**
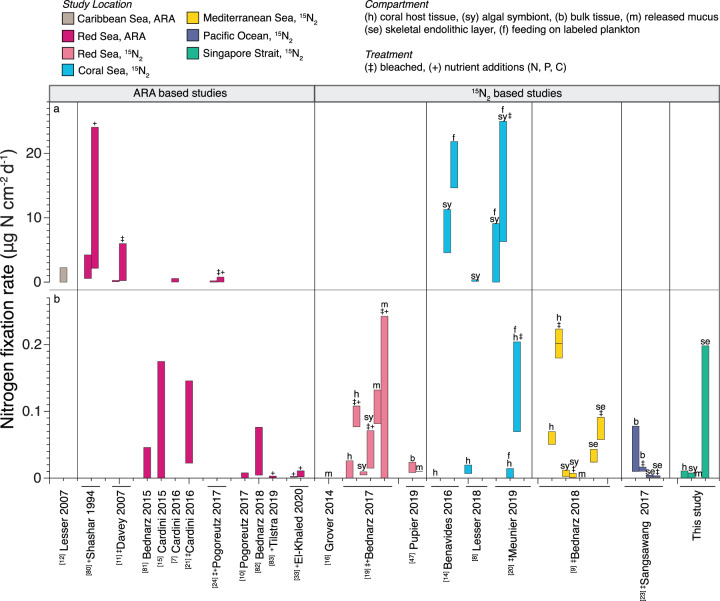
Reported rates of coral-associated N_2_ fixation standardized to µg N cm^−2^ d^−1^. N_2_ fixation rates are split into **a** >0.25 µg N cm^−2^ d^−1^, **b** <0.25 µg N cm^−2^ d^−1^ [7–12, 14–16, 19–21, 23, 24, 33, 47, 78, 109, 114, 115]. The acetylene reduction assay (ARA) measures gross rates of N_2_ fixation in the entire coral holobiont. Studies using isotopic labeling (^15^N_2_) measure net assimilation of fixed nitrogen (N) in the specific tissue compartment analyzed (i.e., m, h, sy, b, f, se). All ARA results were multiplied by 2/3 to convert from moles of C_2_H_4_ to moles of N. Hourly rates were standardized to daily rates. Natural and experimentally manipulated rates are reported separately. (‡) measurements are of bleached coral, and (^+^) measurements are from experiments with manipulated N, P, or C nutrient concentrations.

DDN assimilation rates in coral host and symbiont compartments were within the low end of the range reported elsewhere (Fig. 7). Previous studies have reported up to 6.4-fold higher host DDN assimilation [9] (Fig. 7), as well as a large range in symbiont DDN assimilation from 0.002 µg N cm^−2^ per day [78] to 11 µg N cm^−2^ per day [14] in non-feeding, non-manipulated experiments (Fig. 7). DDN assimilation by released mucus was similar in magnitude to the majority of prior ^15^N_2_ mucus measurements [9, 16, 47], with the exception of Bednarz et al. [19], where mucus DDN assimilation rates were over 200-fold higher than those found in Singapore. Such high rates of mucus fixation could be a result of the dynamic nature of mucus microbial communities, which are influenced by the overlying water column [79, 80]. In Bednarz et al. [19], the water column N:P ratio (1.5) was the lowest reported N:P of all Red Sea studies (Table S1) and 9.7-fold lower than in Singapore, which might have favored water column and mucus-associated diazotrophy.

In contrast with studies from the Coral Sea, which observed significantly higher rates of symbiont DDN assimilation relative to the host [8, 14], we observed slightly higher host than symbiont DDN assimilation, similar to [19, 78], which may be a result of regional and/or species-specific differences, as well as experimental design. As nutrient uptake rates are known to vary between the coral compartments [81, 82], the total incubation time may impact the compartment in which we observe fixed nitrogen. For example, in [19], significant mucus DDN assimilation was detected after 24 h, whereas host and symbiont ^15^N_2_ assimilation was only detected after 72 h. In contrast, some of the highest rates of DDN assimilation were measured in short, day-time experiments [14], and in one experiment, bulk tissue DDN assimilation was greater over a 12-h light period than a 24 h light/dark period [23]. Consequently, variability in the length of coral incubations is likely to cause variability in compartment-specific DDN assimilation results, and ^15^N_2_ incubations of varying durations should be compared with caution. However, longer incubation times alone are unlikely to account for the multiple order of magnitude difference between symbiont DDN assimilation in Singapore and Coral Sea studies. Future work should seek to directly compare rate measurements and microbial communities with standardized methods across reefs.

Most DDN assimilation occurred within the skeletal compartment. While our skeletal DDN assimilation rates were several-fold higher than the two previous studies, these experiments only examined one temperate [78] and one subtropical coral [23] (Fig. 7). As N_2_ fixation measurements using the acetylene reduction assay (ARA) include endolithic fixation, our results suggest that endolithic diazotrophy may explain why ARA typically yields higher rates than ^15^N_2_ incubations (Fig. 7). Moreover, despite high N:P and DIN concentrations at the time of this study, total holobiont DDN assimilation was in the range of previously reported ARA-based measurements. This finding is in agreement with recent work [33] that demonstrated an increase in coral-associated N_2_ fixation in response to eutrophication (Fig. 7). As the endolithic compartment is separated from the overlying water column by the coral tissue and mucus, endolithic microbial activity may be more sensitive to environmental changes in light [81, 83] than changes in nutrient concentrations, allowing high rates of N_2_ fixation activity to persist in eutrophic conditions. While it is unknown whether DDN from the endolithic region is transferred to the overlying coral tissue, experiments have shown that endolithic photoassimilates are transferred to coral tissue, and this transfer increases upon bleaching [5, 84]. In addition, the endolithic community comprises a large percentage of the total holobiont in terms of both biomass and surface area [85–87] (Table 1). Given the high rate of endolithic DDN assimilation, it is possible that endoliths could be a source of nitrogen for coral tissue, or that this fixed nitrogen is incorporated into endolithic photoassimilates, which may be transferred to the coral tissue [5, 84]. While our results cannot provide direct support for this hypothesis, endolithic, host, and symbiont compartments of *Pocillopora* contained fixed nitrogen, yet *nifH* ASVs from Clusters I–III were only found in its skeleton. Additionally, known diazotrophic taxa had higher 16S RNA:DNA-based activity in the skeleton than in the tissue of *Pocillopora*. However, direct evidence is needed to determine the potential role of endolithic-derived nutrients on the overlying coral tissue. Further experimental work, e.g., using isotope pulse-chase methods, would help to directly test the hypothesis that there is exchange of nitrogen between the coral host and endolithic community.

### Trophic position and diazotrophy

Between sites, *Platygyra* showed a high degree of similarity in trophic position and the overall microbial community composition (Figs. 1, 3, and S9), supporting previous findings that corals form species-specific microbial associations [88, 89]. As trends in *Platygyra* DDN assimilation at both sites were also similar between compartments, DDN assimilation may also be highly species-specific. Between species, DDN assimilation rates were not related to coral trophic position, as inferred from our coral isotopic composition, which may explain different hypotheses regarding diazotrophy and trophic position [10, 19]. While *Pocillopora* and *Goniopora* exhibited similar trophic positions and had the same dominant clade of Symbiodiniaceae (Fig. S8), these two corals had drastically different diazotrophic communities, with *Goniopora* having a higher overall abundance of Clusters I–III ASVs, predominately anaerobic Cluster III ASVs. In *Goniopora*, DDN assimilation occurred mainly in the host and endolithic compartments, while in *Pocillopora*, the majority of DDN was assimilated in symbiont and endolithic compartments. These results may be related to morphological differences between the two species, rather than trophic position. Skeletal density and tissue thickness create different oxygen and light gradients, which are known to structure both tissue and endolithic communities [90–92]. Tissue thickness, endolithic biomass, and oxygen consumption were greatest in *Goniopora*, potentially creating more diverse diazotrophic niches. In contrast, *Pocillopora* had the lowest abundance of Cluster I–III *nifH* ASVs, along with the highest oxygen production, thinnest tissue, and lowest endolithic biomass, possibly creating fewer diazotrophic niches. Moreover, mucus-associated N_2_ fixation also differed between both autotrophic species by an average of five-fold. Previous work has proposed that high mucus C:N leads to nitrogen limitation, favoring mucus-associated diazotrophy [47]. While our results follow this trend, with *Pocillopora* having higher released mucus C:N and N_2_ fixation than *Goniopora*, only the C:N of released mucus differed significantly between the two species. Overall, differences in compartment-specific N_2_ fixation rates and diazotrophic community composition between *Pocillopora* and *Goniopora* suggest that diazotrophy in the coral holobiont might not have a straightforward relationship with trophic position, unlike previously suggested [10]. Studies using a larger range of species are needed to determine how coral-associated diazotrophy varies with host physiology.

### DDN budgets

In phosphate- and silicate-replete tropical waters, the water column diazotrophic community is typically comprised of diatom-diazotroph associations [29, 30]. While we found abundant diatom sequences in the seawater and in coral tissue, no ASVs from 16S rRNA and *nifH* seawater samples were related to known diazotroph-associated diatoms [93], and overall, few sequences from Clusters I–III were present. During our study, the water column N:P ratio was ≈14.5, which may explain the low diazotrophic abundance and diversity in the water column [34]. The water column N:P ratio may also drive low rates of mucus-associated diazotrophy, as mucus microbial communities are influenced by the seawater microbial community [79, 80]. Based on budget estimates, neither water column N_2_ fixation nor transfer of DDN to the water column via released mucus were a significant source of new nitrogen production at the time of this study (Table S6).

Despite low rates of N_2_ fixation, we found that DDN might still be a relevant nitrogen source for the coral holobiont. The endolithic community accounts for only 1.4–15% of total holobiont respiration [84, 94, 95] and may have a lower growth rate than the coral tissue. By assuming the same growth rate (and nitrogen demand) for the endolithic community and coral tissue, we may have overestimated overall holobiont nitrogen demand, in which case DDN would have met more than 6% of daily growth demand. However, as little is known about endolithic growth rates in corals, and endoliths need to keep up with skeletal deposition, growth rates may in fact be similar to that of the tissue. Nevertheless, skeletal porewater is rich in dissolved organic nutrients [96, 97], and skeletal nitrogen regeneration by the total endolithic community has been estimated to satisfy 55–65% of daily holobiont N requirements in *M. annularis* [97]. These studies, together with the high rate of endolithic DDN assimilation and higher 16S RNA:DNA ratios of endolithic diazotrophs, suggest that endolith-derived DDN has the potential to be an important source of nitrogen for the coral holobiont.

### Diazotrophic community

The *nifH* ASVs in our corals were predominately from Clusters I, III, and V. The majority of sequences were from Cluster V (∼89% of ASVs), which contains *nifH* homologs involved in chlorophyll biosynthesis that are not known to fix nitrogen (cf. [98, 99]). A similar result was also reported by Lesser et al. [8] using the same primers, which capture a large diversity of *nifH* sequences [56, 100], where ∼89% of ASVs were placed in Cluster V. The majority of our Cluster V sequences were most closely related to chlorophyllide reductase subunit L from *Ostreobium* plastids and Alphaproteobacteria (Rhodobacterales, Rhizobiales), which were all highly abundant in the 16S rRNA microbial community (Figs. S5 and S6). Given the prevalence of Cluster V *nifH* sequences across coral species, analyses of *nifH* diversity or gene copy number that do not differentiate between *nifH* clusters (e.g., [10, 24, 101]) could overestimate diazotrophic diversity and activity in coral. Moreover, taxa containing Cluster V chlorophyllide genes, such as *Ostreobium*, are known to bloom during coral bleaching events [86], which means that particular care must be exercised when analyzing *nifH* abundance in bleaching studies.

Comparison of the *nifH* and 16S rRNA DNA- and RNA-based communities revealed that despite differences in the microbial community between tissue and skeletal compartments, the *nifH* community within a given species was similar between the tissue and skeleton. However, 16S RNA:DNA ratios suggested differences in diazotrophic activity between compartments. In particular, non-heterocystous nitrogen-fixing Cyanobacteria were more abundant and had higher 16S RNA:DNA ratios in the skeleton than the tissue in *Platygyra* and *Pocillopora*, and were also part of the *nifH*-based community in all skeletal samples (Fig. 5). As non-heterocystous Cyanobacteria are commonly found in coral skeletons [102], they may be key members of the coral diazotrophic community. Moreover, as Cyanobacteria can bloom in response to heat stress [103], proliferation of endolithic Cyanobacteria during coral bleaching may explain the increased N_2_ fixation rates measured in multiple experiments [11, 24] (Fig. 7). Future studies should use Cyanobacteria-specific *nifH* probes to accurately quantify their activity, particularly during bleaching. Other potentially important members of the skeletal diazotrophic community included the sulfate-reducing bacteria *Halodesulfovibrio* (Desulfovibrionales), which have been previously isolated from coral endolithic communities [104] and were identified here as relatively active in *Platygyra* and *Pocillopora*; Chloroflexi, which had high 16S RNA:DNA in the skeleton of all species; Gammaproteobacteria (Alteromonadales), particularly in the skeleton of *Pocillopora* and *Goniopora*; and Pseudoxanthobacter (Rhizobiales), which were only identified as relatively active in the skeleton of *Goniopora*. In coral tissue, Rhodospirillales of the family Terasakiellaceae, which contains nitrogen fixers and may be associated with healthy coral tissue [105–108], had high 16S RNA:DNA ratios in the tissue of all species.

Within the RNA-based *nifH* diazotrophic community, the majority of Clusters I–III sequences were comprised of Gammaproteobacteria and Cyanobacteria, whereas Deltaproteobacteria, while present in the DNA-based community, were not abundant (Fig. S14). Across all species, most RNA-based *nifH* ASVs belonged to Cluster I, including *Goniopora*, which had a high abundance of Cluster III diazotrophs in the DNA-based community (Fig. 5). Photosynthetic activity by both Symbiodiniaceae and endolithic algae (e.g., *Ostreobium*) likely favor diazotrophs from Cluster I, which are either aerobic or facultatively anaerobic. In coral tissue, Symbiodiniaceae-derived photosynthates have been proposed as a source of carbon for coral-associated diazotrophs [12, 109]. Correspondingly, we propose that *Ostreobium* may provide a carbon source for endolithic diazotrophs. Although transfer of carbon from Symbiodiniaceae or *Ostreobium* to diazotrophs has yet to be directly documented, such a relationship may explain why some of the highest rates of DDN assimilation reported in the literature were measured in short, day-time experiments [14, 23]. While our incubations lasted 24 h, our *nifH* community composition suggests that the majority of coral-associated diazotrophs are able to tolerate aerobic conditions. However, as incubations were retrieved around midday, our experimental timing may have biased the RNA-based *nifH* community toward Cluster I. While not abundant in the RNA-based community, multiple Cluster III *nifH* ASVs related to sulfate-reduction and sulfur-oxidation were abundant both in the skeleton and tissue (Fig. 5), and 16S RNA:DNA ratios indicated that multiple Deltaproteobacteria taxa were active in the tissue and skeleton. Together, these findings suggest that both compartments are capable of hosting anaerobic N_2_ fixation. Nevertheless, the coexistence of Clusters I and III, even if in the DNA-based community, highlights the heterogeneity of microbial niches in coral skeleton and tissue. Moreover, as the majority of Cyanobacteria were non-heterocystous, their ability to fix nitrogen might depend on temporal or spatial separation from oxygen production.

As a number of potentially diazotrophic Alphaproteobacteria were abundant in the 16S rRNA community, notably Rhizobiales, it is surprising that these taxa were not equally abundant in the *nifH* Clusters I–III sequences. Rhizobiales contains many known diazotrophs that may associate with microalgae [110, 111] and have been found in varying abundance in coral microbial communities [8, 11, 112]. While Rhizobiales have been proposed as possible diazotrophic coral symbionts [112], their role in coral N_2_ fixation is still debated, particularly as some species contain only Cluster V *nifH* homologs and do not appear to fix nitrogen [8, 111]. We found that Rhizobiales had low relative activity in both the tissue and skeleton (16S RNA:DNA <1), and that the majority of Rhizobiales in the *nifH* community had Cluster V *nifH* homologs. Within the *nifH* Clusters I–III community, only Rhizobiales most closely related to Aurantimonadaceae, a family associated with coral white plague disease, displayed signs of activity (16S RNA:DNA = 4.6, Fig. S16). Therefore, despite the abundance of Rhizobiales in our coral samples (especially in the skeleton), it is unlikely that this group was an important part of the diazotrophic community. In future work, more direct techniques, such as RNA stable isotope probing, should be used to validate the taxa-specific trends in relative diazotrophic activity observed here.

While DNA-based microbial community analysis has reshaped our understanding of corals, our knowledge of microbial activity within the coral holobiont is still extremely limited. RNA:DNA ratios are a well-established method for investigating relative microbial activity [39–42], although this method cannot be used as an absolute measure of activity. rRNA gene copy numbers can vary between species and as a function of metabolic state or cell size, and growth rates are not always simply correlated with rRNA concentrations (cf. [42]). To account for these limitations, ratios should be interpreted between related taxonomic groups [40, 42], and RNA-based community composition should be considered as a relative indicator of protein synthesis, rather than an absolute indicator [42]. Despite these considerations, RNA:DNA ratios can provide useful insight into the functionality of coral microbial communities and may be a particularly useful metric during periods of environmental stress.

## Conclusions

Our results demonstrate that while seawater- and mucus-associated N_2_ fixation rates may be low during nutrient-replete conditions, diazotrophs in the coral tissue and skeleton still fix nitrogen on a scale that is relevant to the daily N growth demand of the coral. The majority of RNA-based *nifH* ASVs were from Cluster I (aerobes or facultative anaerobes), supporting the hypothesis that coral-associated diazotrophs tolerate hyperoxic conditions [8, 33]. Given rate discrepancies between incubations of variable length [14, 23], as well as the abundance of Cluster I, we hypothesize that diazotrophy may be linked to photosynthetically derived carbon sources in the tissue and skeleton [12, 109]. Studies at higher spatial and temporal resolution are needed to determine both the carbon source of coral-associated diazotrophs, and the diurnal dynamics of N_2_ fixation and photosynthesis within the holobiont. While Rhizobiales, a proposed mutualistic symbiont, were abundant in the coral microbial community, we found little evidence that they had the capacity for N_2_ fixation. Our results particularly highlight the importance of the endolithic community for coral-associated diazotrophy, and we suggest that the magnitude of endolithic diazotrophy may account for discrepancies between ^15^N_2_- and ARA-based studies. The biogeochemical and physiological significance of the endolithic community is still largely uncharacterized and should be a focus for future research. Between coral species, we found no relationship between trophic position or symbiont clade and DDN assimilation. Instead, physiological differences between species, such as tissue biomass, mucus content, and skeletal density, likely structure the microbial community and create habitats more or less favorable for diazotrophy.

## Supplementary information

Supplementary Information
