# Supplementary material for: Coral-associated nitrogen fixation rates and diazotrophic diversity on a nutrient-replete equatorial reef
Source: ISME J. 2021 Jul 22;16(1):233–46. doi: 10.1038/s41396-021-01054-1 (PMC8692400; doi:10.1038/s41396-021-01054-1)
Supplement: Supplementary file 1 — Supplementary Information [file 41396_2021_1054_MOESM1_ESM.pdf]

# Supplementary Information: Coral-associated nitrogen fixation rates and diazotrophic diversity on a nutrient-replete equatorial reef

**Molly A. Moynihan[1,2*], Nathalie F. Goodkin[2,3,4], Kyle M. Morgan[2], Phyllis Y.Y. Kho[2], Adriana Lopes dos Santos[2], Federico M. Lauro[2,5], David M. Baker[6], Patrick Martin[2]**

## Contents

**1 Methods** **2**
1.1 Incubation set-up . . . 2
1.2 DDN assimilation calculations . . . 2
1.3 Nucleic acid extraction, PCR, sequencing and bioinformatics . . . 3

**2 Tables** **5**
2.1 Reported rates of coral-associated nitrogen fixation . . . 5
2.2 Water Column Characterization . . . 7
2.3 Benthic Cover . . . 8
2.4 Non-enriched isotope results . . . 9
2.5 $^{15}$N excess & $N_2$ fixation rates . . . 10
2.6 Reef-scale $N_2$ fixation budget . . . 11
2.7 16S rRNA alpha diversity . . . 12

**3 Figures** **13**
3.1 Site map . . . 13
3.2 Incubation setup and endolithic layer . . . 14
3.3 Control $\delta^{13}$C versus $\delta^{15}$N . . . 15
3.4 Incubation oxygen concentrations . . . 16
3.5 16S rRNA treemap for DNA- and RNA-based tissue and skeletal communities . . . 17
3.6 16S rRNA relative abundances per sample . . . 18
3.7 16S rRNA plastid sequences . . . 19
3.8 Coral tissue 18S rRNA community relative abundance . . . 20
3.9 18S rRNA PCoA by species and type . . . 21
3.10 18S rRNA non-Symbiodiniaceae treemaps for DNA- and RNA-based tissue and skeletal communities . . . 22
3.11 16S rRNA seawater community treemap . . . 23
3.12 18S and 16S rRNA plastid seawater community treemaps . . . 24
3.13 *nifH* Cluster abundance . . . 25
3.14 *nifH* RNA-based Clusters I–III . . . 26
3.15 Select RNA:DNA ratios of coral 16S rRNA community . . . 27
3.16 RNA:DNA ratios of coral *nifH* community . . . 28

# 1 Methods

## 1.1 Incubation set-up

Due to strong vertical gradients in light at these reefs, all coral fragments were collected and acclimated within ± 1 m depth from their original colonies. Corals were fragmented with a hammer and chisel, attached to a PVC rig using cable ties. The PVC fragmentation rig was coated in food-grade paraffin wax (Gulfwax) to prevent fouling.

Incubation jar lids were modified to incorporate two rubber septa (Wheaton W224100-173) and sealed with silicone, as well as a PVC attachment to hold the coral in place. Corals were retrieved from the reef, placed on new PVC attachments, rinsed briefly with filtered seawater to remove loosely associated organisms, and placed in chambers with filter-sterilized seawater. All incubation chambers were closed while submersed in water to prevent air contamination.

The $^{15}N_2$ label was prepared by degassing 0.22 $\mu$m filtered seawater at 920 mbar for 1 hour [1, 2]. Water was transferred headspace-free to serum bottles (Wheaton 223952) by siphon and crimp-sealed (Wheaton 224187-01) with rubber septa (Wheaton W224100-173). $^{15}N_2$ gas (Cambridge Isotopes NLM-363-1LB, Lot#: I-21065/AR0664729) was added in a ratio of 2ml/100ml to each serum bottle. Serum bottles were inverted during dissolved gas addition and displaced water was captured using a separate outflow needle and syringe. After gas addition, additional filter-sterilized, degassed seawater was injected via the septa to increase pressure in the serum bottle. Serum bottles were shaken for 5 minutes and stored on their sides at room temperature for 24 hours. Final enrichment levels of all serum bottles were >90% $^{15}N_2$. Glassware, tubing, and septa used throughout the experiment were acid-washed with 10% HCl and rinsed throughly with ultrapure water (Elga, 18.2MΩ). More details can be found at protocols.iref.o/researchers/molly-moynihan.

## 1.2 DDN assimilation calculations

To determine diazotroph-derived nitrogen (DDN) assimilation rates, particulate atom % $^{15}$N from paired experimental ($A_e$) and control ($A_c$) replicate samples were subtracted for each fraction (i.e. host tissue, algal symbionts, released mucus, skeleton, and seawater). Results were only considered significant if this difference was greater than $3x$ the standard deviation of repeated $A_c$ measurements, following [3, 4, 5]. Rates below the detection limit were assumed to be zero when calculating average rates, resulting in more conservative estimates. $V(T^{-1})$ is defined as the particulate difference ($A_e - A_c$) divided the amount of dissolved gas label ($A_{15N2} - A_o$) and the incubation time ($\Delta t$) for each chamber (ref. 1). The particulate nitrogen concentration ($[PN]$) was determined from the %N of the experimental sample (ref. 2). Combining these variables, nitrogen fixation rates were calculated using Equation 1 [6] and standardized by dry weight (ref. 3), surface area (ref. 4), and/or volume (refs. 5-6), according to the sample type. $DW_x$ is the dry weight of the specific fraction (host tissue, algal symbionts, released mucus, and seawater particulate organic matter), and $\rho_s$ is the skeletal density (g cm$^{-3}$). Genera-specific density values from Singapore were compiled from Januchowski-Hartley et al. [7]. For coral nitrogen budget calculations, unseparated tissue (host and symbiont) $DW_x$ cm$^{-2}$ was multiplied by %N to determine N content.

$$V(T^{-1}) = \frac{1}{\Delta t}\,\frac{A_e - A_c}{A_{15N2} - A_o} \tag{1}$$

$$[PN]\ (\text{ng g}^{-1}) = \frac{\%N}{100} \times 10^9 \tag{2}$$

$$DDN_{assimilation}\ (\text{ng N g}^{-1}\ \text{DW h}^{-1}) = V(T^{-1}) \times [PN] \tag{3}$$

$$DDN_{assimilation} \text{ (ng N cm}^{-2}\text{ h}^{-1}) = V(T^{-1}) \times [PN] \times \frac{DW_x}{mL} \frac{mL}{cm^2} \tag{4}$$

$$DDN_{assimilation} \text{ (ng N L}^{-1}\text{ h}^{-1}) = V(T^{-1}) \times [PN] \times \frac{DW_x}{L} \tag{5}$$

$$DDN_{assimilation} \text{ (ng N cm}^{-3}\text{ h}^{-1}) = V(T^{-1}) \times [PN] \times \rho_s \tag{6}$$

### 1.3 Nucleic acid extraction, PCR, sequencing and bioinformatics

DNA was extracted from coral tissue and skeletal samples using the DNeasy PowerBiofilm kit (Qiagen 24000-50, Hilden, Germany). The following modifications were made based on Sunagawa et al. [8]: (i) after the addition of lysis solution (FB), 10 $\mu$l of lysozyme (12.5mg/ml) were added to each reaction and samples were incubated at room temperature for 10 minutes; subsequently, (ii) 20 $\mu$l of proteinase-K (20mg/ml) were added and samples were incubated at 65°C for 60 minutes. The remaining protocol followed the manufacturer's instructions. From seawater samples, DNA was extracted from Sterivex filters (1 l seawater) using a modified phenol-chloroform method with the DNeasy PowerSoil kit (Qiagen 12888-50). After $2x$ lysis incubations with solution C1 (70°C, 500rpm, 10 minutes), 700$\mu$l of phenol-chloroform-isoamyl alcohol were added to each reaction (adapted from [9]). Samples were vortexed for 10 minutes and centrifuged at 10,000 $x$ g for 30 seconds. The resulting supernatant was transferred to a clean tube, and remaining steps followed the manufacturer's protocol.

RNA was extracted using Trizol (Invitrogen 15596018, Carlsbad, CA, USA). After phase separation, RNA was precipitated for 10 minutes at room temperature with 250 mL isopropanol, 250 mL high salt solution (0.8 M Na citrate, 1.2 M NaCl), and 1 $\mu$l GlycoBlue coprecipitant (Ambion AM9515, Austin, TX, USA) for every 1 mL of Trizol. After solubilization, RNA was cleaned and concentrated (Zymo, R1017). Samples were checked for DNA contamination by performing PCR with 16S and 18S rRNA primers (see below). cDNA was synthesized from RNA using the SuperScript VILO Master Mix (Invitrogen 11766050) following manufacturer's instructions. Both cDNA and no-reverse transcription controls were amplified, along with extracted DNA, following the conditions below. RNA samples with DNA contamination were treated with ezDNase (Invitrogen 11766051) and re-amplified. Protocols of sample preparation, nucleic acid extractions, and PCR conditions can be found online (protocols.io/researchers/molly-moynihan).

PCR was run in duplicate for all reactions using HotStarTaq (Qiagen 203643) polymerase. All primer sets, with the exception of the nested UNonMet set, contained the following Illumina overhang adapters: 5'-CTTTCCCTACACGACGCTCTTCCGATCT-'3 (forward) and 5'-GGAGTTCAGACGTGTGCTCTTC CGATCT-'3 (reverse). The V6-V8 region of the 16S rRNA gene was amplified with primers B969F '5-ACGCGHNRAACCTTACC-3' and BA1406 5'-ACGGGCRGTGWGTRCAA-3' [10] (c.f. [11, 12, 13]) using the following thermal profile: 95°C for 5 minutes; 30 cycles of 94°C for 1 minute, 52°C for 1 minute, 72°C for 1 minute; and a final cycle of 72°C for 10 minutes. Final primer concentrations were 0.4 mM, the $MgCl_2$ concentration was adjusted to 2 mM, and DMSO was added to achieve a final concentration of 5% v/v. The nitrogenase gene was amplified using universal primers for the nitrogenase iron protein gene (*nifH*), IGK3 5'-GCIWTHTAYGGIAARGGIGGIATHGGIAA-3' and DVV 5'-ATIGCRAAICCICCRCAIACIACRTC-3' [14], with an initial heating step of 95°C for 5 minutes; followed by 35 cycles of 94°C for 1 min, 55°C for 1 minute, 72°C for 1 min; and a final cycle of 72°C for 10 minutes. Final primer concentrations were 0.4 mM, the $MgCl_2$ concentration was adjusted to 4 mM, and BSA was added to a final concentration of 0.1 $\mu$g/$\mu$l. To amplify the V4 region of the 18S rRNA gene, a nested protocol was used to first remove metazoan sequences in coral samples, with the UNonMet primer set: UNonMet-F 5'-GTGCCAGCAGCCGCG-3' and UNonMet-R 5'-TTTAAGTTTCAGCCTTGCG-3' [15], with PCR with settings of 95°C for 5 minutes; 30 cycles of 94°C for 1 min, 51.1°C for 1 minute, 72°C for 1 min; and a final cycle of 72°C for 10 minutes.

PCR products from the UNonMet primer round were cleaned (AMPure XP, Beckman Coulter) and then amplified with 18S rRNA v4 primers, V4-18S-For 5'-CCAGCASCYGCGGTAATTCC-3' and V4-18S-Rev 5'-ACTTTCGTTCTTGATYRATGA-3' [16], with thermocycling conditions of 95°C for 5 minutes; 25 cycles of 94°C for 1 min, 52°C for 1 minute, 72°C for 1 min; and a final cycle of 72°C for 10 minutes. In seawater samples, only V4-18S-For and V4-18S-Rev were used, with the conditions above. Final primer concentrations in both UNonMet and V4-18S reactions were 0.4 mM. Samples were purified, barcoded and sequenced by the GeT-PlaGe platform of GenoToul (INRA Auzeville, Castanet-Tolosan, France) using an Illumina MiSeq platform (2 *x* 250bp).

Per primer set, an average of 43,313 ± 7,400 (16S rRNA), 43,778 ± 14,118 (*nifH*), and 53,148 ± 9,387 (18S rRNA) sequences were generated for each sample. Sequences were trimmed using the cutadapt tool to remove primers, and paired-end reads were filtered with a maximum expected error (maxEE = 5), truncated with the parameter truncQ = 2, and low-quality tails removed (trunLen=(225, 215); 16S rRNA, 18S rRNA) and (trunLen=(220, 215); *nifH*) . Variants were inferred using the DADA2 method [17]. Chimeras were removed from the dataset. Following removal of low-quality sequences, 5,187,478 sequences were retained, of which 1,748,960 were in the 16S rRNA dataset, 980,764 were in the *nifH* dataset, and 2,457,754 in the 18S rDNA dataset. Taxonomic assignment was done with a Baysian classifier, using the SILVA 16S rRNA database (v138) [18, 19], PR$^2$ database (v4.12) [20], and the *nifH* DADA2 database [21]. After taxonomic assignment, class and order ranks were standardized between the SILVA and *nifH* DADA2 ASV tables to facilitate comparison, based on NCBI taxonomy.

Negative controls were sequenced and used to control for contamination using the prevalence method of decontam with a threshold of 0.1 [22]. Additional seawater samples, processed together with the seawater samples used in this study, were included in the decontam step to avoid bias in identifying contaminants. In the 16S rRNA dataset, 22 ASVs were identified by decontam and removed. No contaminating sequences were identified in the 18S rRNA and *nifH* datasets.

A total of 13,047 non-plastid amplicon sequence variants (ASVs) were found in the 16S rRNA analysis (with 572 plastid ASVs), 3,632 *nifH* ASVs, and 1,031 18S rRNA ASVs. In seawater samples, 16S rRNA, 16S rRNA plastid, *nifH*, and 18S rRNA ASV totals were 407, 34, 340, and 382, respectively. With the exception of alpha diversity calculations, ASVs with fewer than 10 occurrences were excluded from analyses. Plastid ASVs were removed from the 16S rRNA community analysis. For all amplicon datasets, replicate sample dispersion displayed homogeneity (Betadisper, $p > 0.05$).

The *nifH* phylogeny was made using reference sequences from Raymond et al. [23], Meheust et al. [24] and Heller et al. [25]. Cluster definitions were based on Meheust et al. [24]. The representative sequences of *nifH* ASVs (3,632) were translated into protein sequences using Geneious Prime 2019.2.3 with standard genetic code (https://www.geneious.com), and correct reading frames were checked manually for all sequences. Sequence alignment was performed with the MAFFT Alignment plugin with the following settings: auto algorithm, BLOMSUM62 scoring matrix, a gap open penalty of 1.53, and an offset value of 0.123. Cluster assignment done manually by comparing groups of aligned ASVs (≈500 ASVs) with reference sequences [23, 24]. In total, 5.08% of ASVs belonged to Cluster I, 0.66% to Cluster II, 4.15% to Cluster III, 1.11% to Cluster IV, 88.91% to Cluster V, 0.01% to Cluster VI, and 0.08% were not assigned a cluster. To make the final tree (Fig. 4), all Cluster I – III ASVs from coral DNA- and RNA- based samples were added to the tree, as well as ASVs from Clusters VI-VI with a normalized abundance ≥ 200 for each species, site, and sample type (i.e. tissue or skeleton), respectively. The tree was assembled using the FastTree plugin. Both FASTA formatted sequence alignment and Newick files can be found on https://github.com/moyn413/Singapore-coral-microbes.

# 2 Tables

## 2.1 Reported rates of coral-associated nitrogen fixation

Table S1: Rate measurements of nitrogen fixation in corals using the acetylene reduction assay (ARA), $^{15}N_2$ bubble method, or $^{15}N_2$ dissolution method. The $^{15}N_2$ bubble method underestimates rates due to a delay $^{15}N_2$ in equilibration [1]. Dissolved inorganic nitrogen (DIN), nitrate ($NO_3$), dissolved inorganic phosphorus (DIP) concentrations, and the ratio of DIN to DIP are listed, where reported. For [26], only total dissolved nitrogen and phosphorus (TDN, TDP) were reported. ($^\bullet$) Denotes accompanying *nifH* metabarcoding and ($^\diamond$) denotes *nifH* copy number analysis. (*) Indicates subsampling within the total incubation time (h), ($^+$) indicates an artificial nutrient addition, and (‡) indicates bleached coral.

| **Study** | **Method** | **Time (h)** | **Rate** | **Location** | **Set-up** | **Dissolved Nutrients** | **DIN:DIP** | **Coral Species** |
|---|---|---|---|---|---|---|---|---|
| Shashar et al. 1994 [27] | ARA | 60 | 0.0 – 18.81 nmol $C_2H_2$ $cm^{-2}$ $h^{-1}$ (*in-situ*)<br><br>9.6 – 107.4 nmol $C_2H_2$ $cm^{-2}$ $h^{-1}$ (*F. favus*; *aquaria, light, + glucose, + PSII inhibition*)<br><br>0.7 – 56.4 nmol $C_2H_2$ $cm^{-2}$ $h^{-1}$ (*F. favus* skeleton; *aquaria, light, + glucose*) | Red Sea | *in situ* & aquaria | NR | NR | *Acropora*<br>*S. pistillata*<br>*C. chalcidicum*<br>*F. favus*<br>*P. lamellina*<br>*P. lobata*<br>*P. damicornis* (*in situ*)<br>*F. favus* (aquaria) |
| Lesser et al. 2007 [28] | ARA | 24* | 0 – 10 nmol $C_2H_2$ $cm^{-2}$ $h^{-1}$ | Bahamas | aquaria | NR | NR | *M. cavernosa* |
| Davey et al. 2007 [29] | ARA | 6* | 0.30 – 1.16 nmol $C_2H_2$ $cm^{-2}$ $h^{-1}$ (live coral)<br>1.08 – 26.66 nmol $C_2H_2$ $cm^{-2}$ $h^{-1}$ (dead coral ‡) | Great Barrier Reef | aquaria | NR | NR | *A. aspera* |
| Grover et al. 2014 [30] | $^{15}N_2$ bubble | 24 | 0.08 – 0.32 ng N $cm^{-2}$ $day^{-1}$ (mucus) | Red Sea | aquaria | nM concentrations of $NO_3$, $NH_4$, DIP | NR | *S. pistillata*<br>*C. gracilis*<br>*Porites sp.* |
| Bednarz et al. 2015 [31] | ARA | 24* | 0.001 – 0.205 nmol $C_2H_2$ $cm^{-2}$ $h^{-1}$ | Red Sea | aquaria | [$NH_4^+$] = 0.14 – 0.46$\mu$M<br>[DIP] = 0.04 – 0.11$\mu$M | NR | *Sarcophyton*<br>*Xeniidae* |
| Cardini et al. 2015 [32] | ARA | 24* | 0 – 0.78 nmol $C_2H_2$ $cm^{-2}$ $h^{-1}$ | Red Sea | aquaria | [DIN] = 0.17 – 1.03 $\mu$M<br>[DIP] = 0.04 – 0.1$\mu$M | 4.25 – 10.75 | *Acropora*<br>*Pocillopora*<br>*Stylophora*<br>*Goniastrea* |
| Cardini et al. 2016 [33] | ARA | 24* | 0.1 – 2.5 nmol $C_2H_2$ $cm^{-2}$ $h^{-1}$ (hard coral)<br>0.1 – 0.65 nmol $C_2H_2$ $cm^{-2}$ $h^{-1}$ (soft coral) | Red Sea | aquaria | [DIN] = 0.31 – 1.25$\mu$M<br>[DIP] = 0.04 – 0.11$\mu$M | 7.7 – 11.7 | *Acropora*<br>*Pocillopora*<br>*Stylophora*<br>*Goniastrea*<br>Xeniidae<br>*Sarcophyton* |
| Cardini et al. 2016 [34] | ARA | 6 (D)*<br>6 (N)* | 0.18 – 0.65 nmol $C_2H_2$ $cm^{-2}$ $h^{-1}$ (*light* ‡)<br>0.1 – 0.4 nmol $C_2H_2$ $cm^{-2}$ $h^{-1}$ (*dark* ‡) | Red Sea | aquaria | [DIN] = 0.41 – 0.45 $\mu$M<br>[DIP] = 0.02 – 0.03 $\mu$M | 15 – 22.5 | *S. pistillata*<br>*A. hemprichii* |
| Benavides et al. 2016 [35] | dissolved $^{15}N_2$ | 4 | 190 – 470 ng N $cm^{-2}$ $h^{-1}$ (symbiont)<br>610 – 910 ng N $cm^{-2}$ $h^{-1}$ (symbiont, with labelled plankton) | New Caledonia | aquaria | NR | NR | *S. pistillata* |
| Pogoreutz et al. 2017 [26] | ARA$^\diamond$ | 24* | 0 – 0.9 nmol $C_2H_2$ $cm^{-2}$ $h^{-1}$<br>0.1 – 3.5 nmol $C_2H_2$ $cm^{-2}$ $h^{-1}$ ($^+$DOC ‡) | Red Sea | aquaria | [TDN] = 20$\mu$M<br>[TDP] = 0.28$\mu$M<br>$^+$ [DOC] = 150 – 1600 | NR | *P. verrucosa* |
| Pogoreutz et al. 2017 [36] | ARA$^\diamond$ | 24 | 0 – 0.035 nmol $C_2H_2$ $cm^{-2}$ $h^{-1}$ | Red Sea | aquaria | [DIN] ≤ 0.6$\mu$M<br>[TP] ≤ 0.3$\mu$M | NR | *P. verrucosa*<br>*S. pistillata*<br>*P. granulosa*<br>*C. echinata* |
| Sangsawang et al. 2017 [37] | $^{15}N_2$ bubble | 12, 24 | 0.42 – 3.24 ng N $cm^{-2}$ $h^{-1}$ (bulk tissue)<br>0.43 – 0.69 ng N $cm^{-2}$ $h^{-1}$ (bulk tissue ‡)<br>0.05 – 0.18 ng N $cm^{-2}$ $h^{-1}$ (skeleton)<br>0.05 – 0.15 ng N $cm^{-2}$ $h^{-1}$ (skeleton ‡) | Okinawa | aquaria acclimated, incubated on reef | NR | NR | *Porites lutea* |
| Bednarz et al. 2017 [3] | dissolved $^{15}N_2$ | 24, 72 | 3.4 – 5.5 ng N $cm^{-2}$ $h^{-1}$ (mucus)<br>9.9 – 10.1 ng N $cm^{-2}$ $h^{-1}$ (mucus ‡)<br>0 ng N $cm^{-2}$ $h^{-1}$ (mucus $^+$P)<br>0 – 1.08 ng N $cm^{-2}$ $h^{-1}$ (host)<br>4.3 – 4.5 ng N $cm^{-2}$ $h^{-1}$ (host ‡)<br>3.2 – 3.9 ng N $cm^{-2}$ $h^{-1}$ (host $^+$P)<br>0.2 – 0.4 ng N $cm^{-2}$ $h^{-1}$ (symbiont)<br>0.62 – 1.48 ng N $cm^{-2}$ $h^{-1}$ (symbiont ‡)<br>1.64 – 2.96 ng N $cm^{-2}$ $h^{-1}$ (symbiont$^+$P) | Red Sea | aquaria | [DIN] = 0.51$\mu$M<br>[DIP] = 0.34$\mu$M<br>$^+$[DIP] = 3$\mu$M | 1.5<br>$^+$0.17 | *S. pistillata* (shallow) |

| **Paper** | **Method** | **Time (h)** | **Rate** | **Location** | **Set-up** | **Dissolved Nutrients** | **DIN:DIP** | **Coral Species** |
|---|---|---|---|---|---|---|---|---|
| Bednarz et al. 2018 [38] | ARA | 24 | 0.02 – 0.34 nmol $C_2H_2$ $cm^{-2}$ $h^{-1}$ | Red Sea | aquaria | [DIN] = 0.19; 0.75<br>[DIP] = 0.012; 0.045 | 15.8 – 16.7 | *S. pistillata* |
| Bednarz et al. 2018 [39] | dissolved $^{15}N_2$ • | 24 | 2.1 – 2.9 ng N $cm^{-2}$ $h^{-1}$ (host)<br>7.5 – 9.3 ng N $cm^{-2}$ $h^{-1}$ (host ‡)<br>0.1 – 0.5 ng N $cm^{-2}$ $h^{-1}$ (symbiont)<br>0.05 – 0.3 ng N $cm^{-2}$ $h^{-1}$ (symbiont ‡)<br>1.0 – 1.8 ng N $cm^{-2}$ $h^{-1}$ (skeleton)<br>2.4 – 3.8 ng N $cm^{-2}$ $h^{-1}$ (skeleton ‡) | Mediterranean | aquaria | $[NO_3] = 0.5\mu M$<br>$[DIP] = 0.1\mu M$ | NR | *O. patagonica* |
| Lesser et al. 2018 [40] | dissolved $^{15}N_2$ • | 2.0–2.5 | 5.7 – 16.3 ng N $cm^{-2}$ $h^{-1}$ (95% in symbiont tissue) | Great Barrier Reef | seawater table | $[NOx] = 0.71\mu M$ | NR | *S. pistillata* |
| Pupier et al. 2019 [5] | dissolved $^{15}N_2$ | 24 | 1.1 – 8.7 ng N $L^{-1}$ $h^{-1}$ / 0.42 ng N $cm^{-2}$ $h^{-1}$ (mucus)<br>0.02 – 0.3 ng N mg $DW^{-1}$ $h^{-1}$ (symbiont)<br>0.1 – 0.45 ng N mg $DW^{-1}$ $h^{-1}$ (host)<br>0.35 – 0.99 ng N $cm^{-2}$ $h^{-1}$ (total assimilation) | Red Sea | aquaria | $[DIN] < 0.5\mu M$<br>$[DIP] < 0.2\mu M$ | NR | *A. eurystoma*<br>*P. damicornis*<br>*Goniastrea*<br>*Cynaria* |
| Tilstra et al. 2019 [41] | ARA ◦ | 24 | 0.0025 – 0.014 nmol $C_2H_2$ $cm^{-2}$ $h^{-1}$ ($^+$P, $^+$N) | Red Sea | aquaria | $^+[NO_3]$ ~ 3 μM<br>$^+[DIP]$ ~ 0.4μM | NR | *A. hemprichii*<br>*P. granulosa*<br>*M. dichotoma* |
| Meunier et al. 2019 [42] | dissolved $^{15}N_2$ | 24 | 0 –0.3795 μg N $cm^{-2}$ $h^{-1}$(symbiont, with labelled plankton)<br>0.2622 – 1.0402 μg N $cm^{-2}$ $h^{-1}$(‡ symbiont, with labelled plankton)<br>0 – 0.0006 μg N $cm^{-2}$ $h^{-1}$(host, with labelled plankton)<br>0.0029 – 0.0085 μg N $cm^{-2}$ $h^{-1}$ (‡ host, with labelled plankton) | New Caledonia | aquaria | NR | NR | *S. pistillata* |
| El-Khaled et al. 2020 [43] | ARA | 24 | 0.007 – 0.011 nmol $C_2H_2$ $cm^{-2}$ $h^{-1}$ ($^+$ $NO_3$ incubated)<br>0.008 – 0.048 nmol $C_2H_2$ $cm^{-2}$ $h^{-1}$ ($^+$ eutrophied in situ, $^+$ $NO_3$ incubated) | Red Sea | in situ eutrophication, aquarium incubation | In situ:<br>$[DIN] = 0.4\mu M$ (control)<br>$[DIN] = 1.4\mu M$ (eutrophied)<br>Incubations:<br>$[DIN] = 5\mu M$ | 8 (control)<br>10 (eutrophied)<br>> 30 (incubation) | *P. verrucosa* |

## 2.2 Water Column Characterization

Table S2: Environmental parameters during the study period at Hantu and Kusu reefs. Dissolved nutrient concentrations are reported in $\mu$mol $L^{-1}$, with dissolved inorganic nitrogen (DIN) as the sum of $[NO_x]$ and $[NH_4^+]$ and N:P as the ratio of DIN to dissolved inorganic phosphorus (DIP) (i.e. phosphate). Samples were collected at 5 m depth. Temperature, salinity, and chlorophyll-a were measured at the experimental site using a CTD profiler at 5 m depth. The daily light integral (DLI) (mol photons $m^{-2}$ $d^{-1}$) of photosynthetically active radiation (PAR) was measured using loggers deployed approximately 1 m above the incubation site (3-4 m depth) [44]. ($^\star$) denotes the date water was collected for filter-sterilization, ($^\circ$) denotes the date water was sampled for water column incubations and that filter-sterilized water was used in coral incubations.

| **Site** | **Date** | **Salinity (psu)** | **Temperature (°C)** | **DIN ($\mu$mol $L^{-1}$)** | **DIP ($\mu$mol $L^{-1}$)** | **N:P** | **Silicate ($\mu$mol $L^{-1}$)** | **Chlorphyll a ($\mu$g $L^{-1}$)** | **PAR DLI (mol photons $m^{-2}$ $d^{-1}$)** |
|---|---|---|---|---|---|---|---|---|---|
| Hantu | 30/7/18 $^\star$ | 29.6 | 29.5 | 2.80 | 0.22 | 13.02 | 8.80 | 0.53 | - |
| Hantu | 7/8/18 $^\circ$ | 29.0 | 29.3 | 3.04 | 0.21 | 14.63 | 11.37 | 0.97 | 12.08 |
| Kusu | 2/8/18 $^\star$ | 30.1 | 29.4 | 3.48 | 0.23 | 15.32 | 7.36 | 0.93 | - |
| Kusu | 13/8/18 $^\circ$ | 29.9 | 29.5 | 3.25 | 0.21 | 15.24 | 8.73 | 0.60 | 19.02 |

### 2.3 Benthic Cover

Table S3: Select benthic cover data from Hantu and Kusu reefs compiled from [45] and [7]. Hard coral diversity reported only includes zooxanthellate Scleractinian species [46].

| | **Hantu** | **Kusu** |
|---|---|---|
| **Benthic Substrate** | **% Cover** | |
| Live hard coral | 39.1 ± 4.8 | 52.8 ± 6.1 |
| *Goniopora* | 12.33 ± 21.98 | 0.62 ± 1.17 |
| *Pocillopora* | 0 | 1.13 ± 2.78 |
| *Platygyra* | 2.39 ± 3.08 | 1.2 ± 1.42 |
| Macroalgae | 16.9 ± 3.8 | 5.2 ± 2.2 |
| Sponge | 3.0 ± 0.7 | 1.6 ± 0.5 |
| Soft coral | 0 | 2.3 ± 0.9 |
| **Diversity** | **# Species** | |
| Hard coral | 73 | 91 |

## 2.4 Non-enriched isotope results

Table S4: $\delta^{15}$N isotope data of baseline and control coral fragments from Hantu and Kusu reefs. Baseline samples were collected directly from the reef. Control samples were fragmented and acclimated for one week on the reef, and then incubated with no isotope label. Water column particulate organic matter (POM) $\delta^{15}$N was sampled on the day of incubations.

| Site | Sample | Type | Baseline $\delta^{15}$N | Control $\delta^{15}$N | Control – Baseline $\delta^{15}$N |
|---|---|---|---|---|---|
| Hantu | *Goniopora* | Homogenate | 6.70 ± 0.14 | 6.79 ± 0.13 | 0.09 ± 0.19 |
| | | Host | 6.70 ± 0.33 | 6.63 ± 0.18 | -0.07 ± 0.38 |
| | | Symbiont | 6.66 ± 0.11 | 6.77 ± 0.31 | 0.12 ± 0.33 |
| | *Platygyra* | Homogenate | 6.79 ± 0.37 | 7.0 ± 0.24 | 0.21 ± 0.45 |
| | | Host | 8.10 ± 0.26 | 7.98 ± 0.61 | -0.12 ± 0.66 |
| | | Symbiont | 6.53 ± 0.25 | 6.42 ± 0.34 | -0.10 ± 0.42 |
| | Water Column | POM | – | 3.49 | – |
| Kusu | *Platygyra* | Homogenate | 7.06 ± 0.46 | 7.68 ± 0.71 | 0.63 ± 0.84 |
| | | Host | 8.01 ± 0.63 | 8.21 ± 0.37 | 0.19 ± 0.73 |
| | | Symbiont | 6.31 ± 0.26 | 6.57 ± 0.36 | 0.26 ± 0.44 |
| | *Pocillopora* | Homogenate | 7.14 ± 0.35 | 7.51 ± 0.26 | 0.37 ± 0.44 |
| | | Host | 7.65 ± 0.14 | 7.60 ± 0.19 | -0.05 ± 0.24 |
| | | Symbiont | 6.31 ± 0.62 | 7.23 ± 0.83 | 0.92 ± 1.04 |
| | Water Column | POM | – | 4.30 | – |

## 2.5 $^{15}N$ excess & $N_2$ fixation rates

Table S5: $^{15}N$ excess between atom% $^{15}N$ of labelled and unlabelled samples ($A_e - A_c$) (ref 1). Rates were defined as detectable when ($A_e - A_c$) was greater than 3× the standard deviation of repeated control measurements ([3, 4]). Rates below the detection limit were considered to be zero when determining replicate averages. Here, rates are also presented only considering rates above the detection limit ($^{15}N$ excess >0).

| | **Coral Compartment** | **Average $^{15}N$ excess** | **n** | **ng N $cm^{-2}$ $h^{-1}$** | **Average $^{15}N$ excess (>0)** | **n** | **ng N $cm^{-2}$ $h^{-1}$** |
|---|---|---|---|---|---|---|---|
| Hantu-*Goniopora* | Skeleton | 0.00029 | 3 | 3.278 ± 4.385 | 0.00043 | 2 | 4.916 ± 4.727 |
| | Mucus | 0.00005 | 4 | 0.0005 ± 0.0009 | 0.00019 | 1 | 0.002 |
| | Host | 0.00004 | 4 | 0.113 ± 0.226 | 0.00016 | 1 | 0.451 |
| | Symbiont | 0 | 4 | 0 | 0 | 0 | 0 |
| Hantu-*Platygyra* | Skeleton | 0.00044 | 3 | 3.306 ± 3.140 | 0.00066 | 2 | 4.959 ± 1.825 |
| | Mucus | 0.00076 | 4 | 0.007 ± 0.010 | 0.00101 | 3 | 0.010 ± 0.011 |
| | Host | 0.00012 | 4 | 0.040 ± 0.079 | 0.00047 | 1 | 0.158 |
| | Symbiont | 0.00006 | 4 | 0.062 ± 0.125 | 0.00026 | 1 | 0.249 |
| Kusu-*Platygyra* | Skeleton | 0.00039 | 4 | 3.502 ± 4.049 | 0.00078 | 2 | 7.003 ± 0.381 |
| | Mucus | 0.00031 | 4 | 0.006 ± 0.008 | 0.00063 | 2 | 0.013 ± 0.005 |
| | Host | 0.00016 | 4 | 0.061 ± 0.081 | 0.00031 | 2 | 0.122 ± 0.069 |
| | Symbiont | 0.00010 | 4 | 0.139 ± 0.164 | 0.00020 | 2 | 0.277 ± 0.061 |
| Kusu-*Pocillopora* | Skeleton | 0.00053 | 4 | 2.858 ± 2.557 | 0.00053 | 4 | 2.858 ± 2.557 |
| | Mucus | 0.00007 | 3 | 0.005 ± 0.009 | 0.00021 | 1 | 0.016 |
| | Host | 0.00013 | 4 | 0.050 ± 0.101 | 0.00051 | 1 | 0.201 |
| | Symbiont | 0.00028 | 4 | 0.166 ± 0.117 | 0.00038 | 3 | 0.221 ± 0.048 |

## 2.6 Reef-scale $N_2$ fixation budget

Table S6: Reef-scale estimations of $N_2$ fixation biogeochemical significance. Reef surface area was estimated using Google Earth and multiplied by an average water column depth of 5 m to determine the water column volume over the reef. Coral cover was measured by Bauman et al. [45]. Effective hard coral surface area was estimated by multiplying the surface area by 2.2 to account for macro-scale rugosity [7] and by 4 to account for micro-scale rugosity (effective coral surface area) [47]. Released mucus-associated and water column $N_2$ fixation rates are based on data in this study. DIN at each site is provided in the table for biogeochemical context. Calculations for Hantu reef are provided below in Equations 7 and 8.

| | **Hantu** | **Kusu** |
|---|---|---|
| DIN ($\mu$mol L$^{-1}$) | 2.90 | 3.37 |
| Planar reef area (m$^2$) | $2.80x10^4$ | $2.80x10^4$ |
| Water column volume (l reef$^{-1}$) | $1.40x10^8$ | $1.40x10^8$ |
| Percent hard coral cover (%) | 40% | 53% |
| Coral planar surface area (cm$^{-2}$ reef$^{-1}$) | $1.12x10^8$ | $1.48x10^8$ |
| Effective coral surface area (cm$^{-2}$ reef$^{-1}$) | $9.85x10^8$ | $1.30x10^9$ |
| Released mucus $N_2$ fixation (ng N cm$^2$ h$^{-1}$) | 0.005 | 0.005 |
| Released mucus $N_2$ fixation (nmol N d$^{-1}$ reef$^{-1}$) | $8.44x10^6$ | $1.12x10^7$ |
| Released mucus $N_2$ fixation (nmol l$^{-1}$ d$^{-1}$) | 0.06 | 0.08 |
| Water column $N_2$ fixation (ng N l$^{-1}$ h$^{-1}$) | 0.08 | 0.07 |
| Water column $N_2$ fixation (nmol N l$^{-1}$ d$^{-1}$) | 0.14 | 0.12 |
| Released mucus $N_2$ fixation relative to water column $N_2$ fixation (%) | 44.0 | 66.6 |

**Hantu Calculations:**

$$\text{Released mucus N}_2\text{ fixation day}^{-1} = \frac{0.005ngN}{cm^2h}\times\frac{nmol}{14ng}\times\frac{24h}{d}\times\frac{9.85x10^8cm^2}{reef}\times\frac{reef}{1.4x10^8l} = 0.06\ nmol\ N\ l^{-1}d^{-1} \quad (7)$$

$$\text{Seawater N}_2\text{ fixation day}^{-1} = \frac{0.08ngN}{l\ h}\times\frac{nmol}{14ng}\times\frac{24h}{d} = 0.14\ nmol\ N\ l^{-1}d^{-1} \quad (8)$$

## 2.7 16S rRNA alpha diversity

Table S7: Number of observed ASVs and alpha diversity indices (Shannon, Simpson, Chao1) for 16S rRNA DNA- and RNA- based communities for of the coral tissue and skeleton from each species at Hantu and Kusu reefs, along with the DNA-based seawater community. The number of replicates (n) are reported along with the standard deviation.

| | Site | Sample | Type | Observed | Shannon | Simpson | Chao1 | n |
|---|---|---|---|---|---|---|---|---|
| DNA | Hantu | *Platygyra* | Tissue | 649.5 ± 119.98 | 5.16 ± 0.46 | 0.98 ± 0.01 | 674 ± 121.82 | 4 |
| | | | Skeleton | 500.75 ± 69.35 | 4.86 ± 0.57 | 0.97 ± 0.03 | 527.35 ± 78.1 | 4 |
| | | *Goniopora* | Tissue | 304.75 ± 71.63 | 3.67 ± 0.57 | 0.89 ± 0.07 | 308.98 ± 70.35 | 4 |
| | | | Skeleton | 166.5 ± 38.84 | 2.7 ± 0.48 | 0.79 ± 0.08 | 172.52 ± 37.72 | 4 |
| | | Seawater | | 312 | 4.63 | 0.74 | 383.75 | 1 |
| | Kusu | *Pocillopora* | Tissue | 303 ± 61.36 | 4.03 ± 0.13 | 0.94 ± 0.01 | 311.74 ± 65.46 | 4 |
| | | | Skeleton | 142.5 ± 74.74 | 3.22 ± 0.74 | 0.87 ± 0.11 | 146.02 ± 74.76 | 4 |
| | | *Platygyra* | Tissue | 641.5 ± 114.07 | 5.43 ± 0.28 | 0.99 ± 0 | 670.4 ± 110.88 | 4 |
| | | | Skeleton | 468.25 ± 121.66 | 4.85 ± 0.22 | 0.98 ± 0 | 486.52 ± 133.26 | 4 |
| | | Seawater | | 336 | 4.66 | 0.98 | 360.24 | 1 |
| RNA | Hantu | *Platygyra* | Tissue | 526.75 ± 272.61 | 4.93 ± 0.74 | 0.96 ± 0.03 | 565.47 ± 292.33 | 4 |
| | | | Skeleton | 457.67 ± 214.57 | 4.47 ± 0.48 | 0.94 ± 0.05 | 473.34 ± 219.65 | 3 |
| | | *Goniopora* | Tissue | 223.5 ± 96.32 | 3.07 ± 1.37 | 0.78 ± 0.22 | 236.71 ± 108.39 | 4 |
| | | | Skeleton | 252.5 ± 70 | 3.62 ± 0.19 | 0.91 ± 0 | 272.63 ± 63.93 | 2 |
| | Kusu | *Pocillopora* | Tissue | 223 ± 55.05 | 3.27 ± 0.58 | 0.86 ± 0.09 | 229.5 ± 56.56 | 4 |
| | | | Skeleton | 109.5 ± 76.7 | 3.17 ± 0.64 | 0.9 ± 0.06 | 109.5 ± 76.7 | 4 |
| | | *Platygyra* | Tissue | 675.5 ± 188.86 | 5.51 ± 0.39 | 0.99 ± 0.01 | 730.45 ± 181.12 | 4 |
| | | | Skeleton | 386.25 ± 97.28 | 4.49 ± 0.46 | 0.96 ± 0.02 | 435.85 ± 101.69 | 4 |

# 3 Figures

## 3.1 Site map

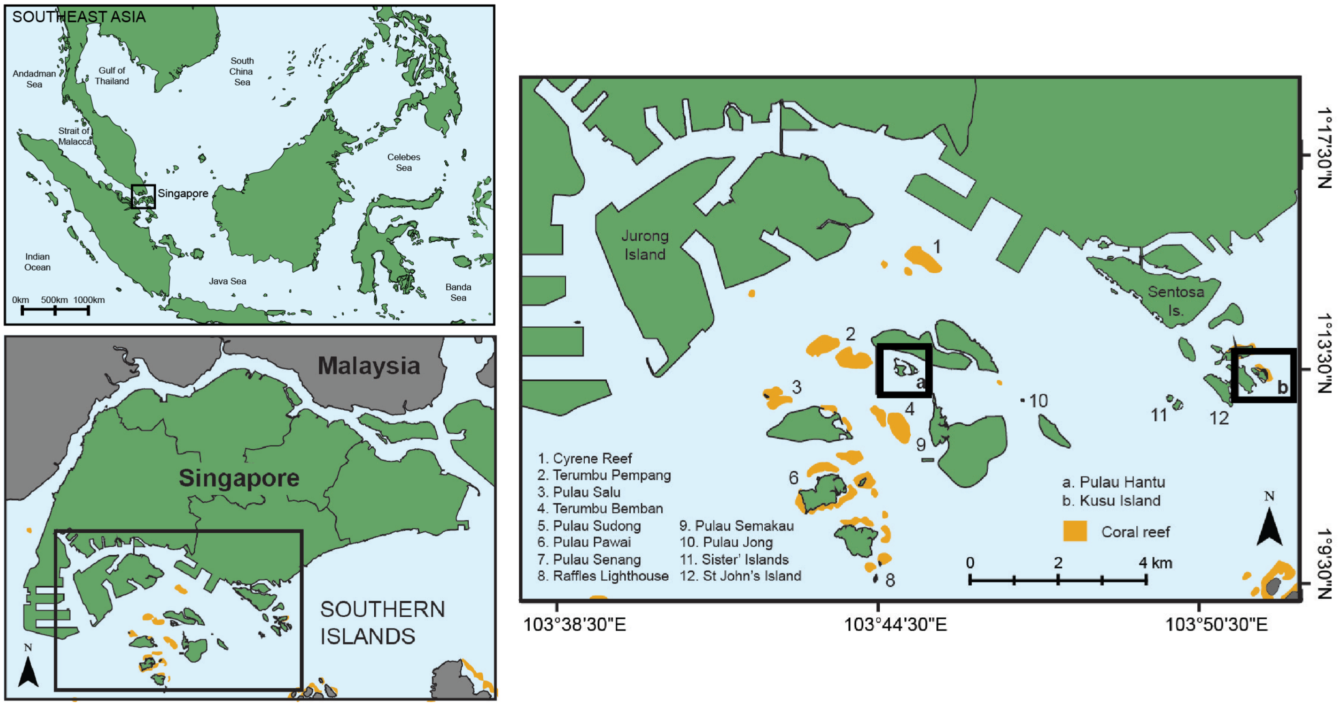


Figure S1: Location of sampling sites, Pulau Hantu and Kusu Island, within the Southern Islands of Singapore. Sites are separated by approximately 13 km. Corals were fragmented, acclimated, and incubated at ~ 5 m depth on the western reef at Pulau Hantu and the eastern reef at Kusu Island. Site map from [44].

## 3.2 Incubation setup and endolithic layer

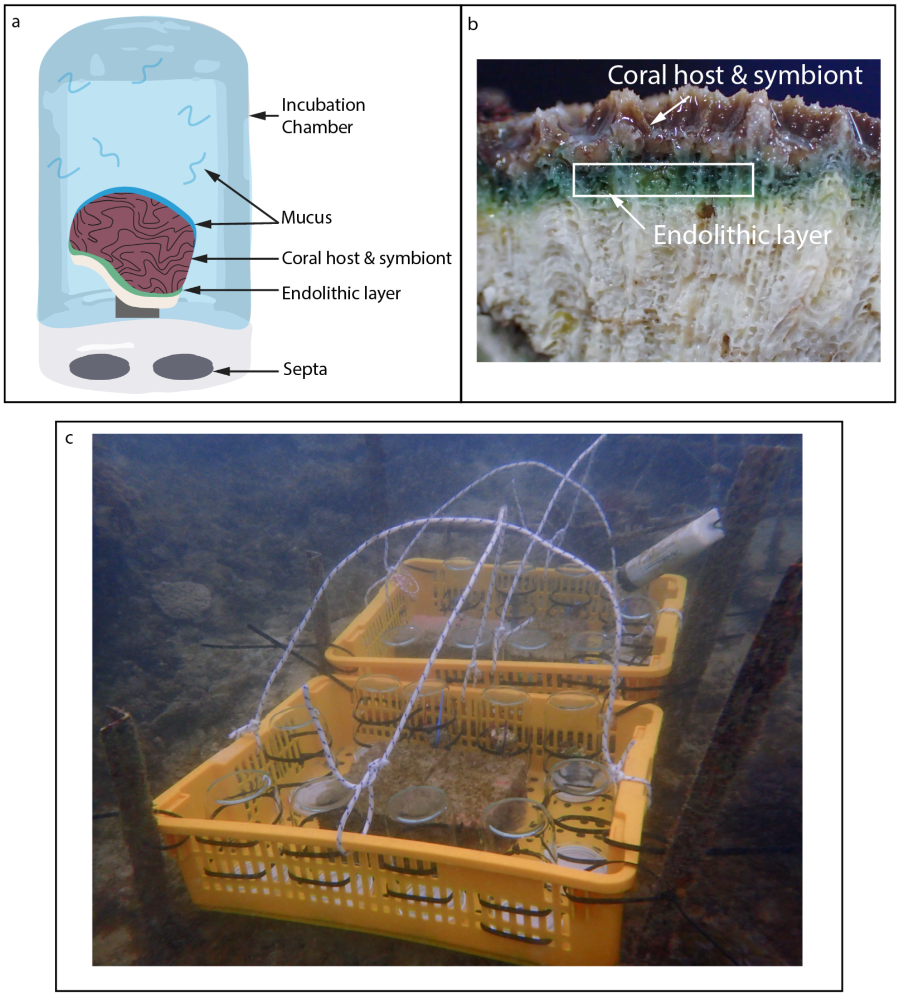


Figure S2: (a) Incubation jar modified to include septa for $^{15}N_2$ injection. Released mucus was collected by filtering water in the jar at the end of the experiment. Coral host and symbiont tissue were removed by airbrushing, and subsequently, the upper portion of the skeleton was separated by hand drill or chisel. (b) Cross section of a *Platygyra* fragment showing the endolithic layer (here, green in color). (c) Incubation experiment deployed on the reef.

### 3.3 Control $\delta^{13}C$ versus $\delta^{15}N$

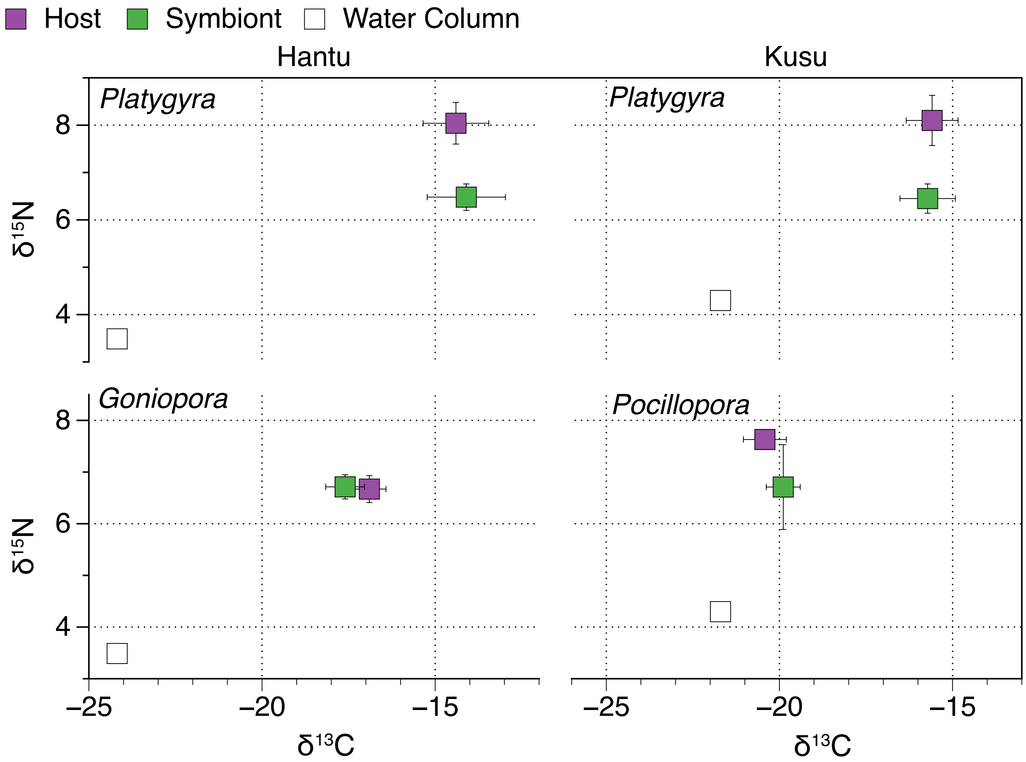


Figure S3: Average $\delta^{13}C$ versus $\delta^{15}N$ of both baseline and control samples for host tissue and algal symbiont fractions, along with water column particulate matter from each reef, collected on GF/F filters (0.7 µm pore size).

## 3.4 Incubation oxygen concentrations

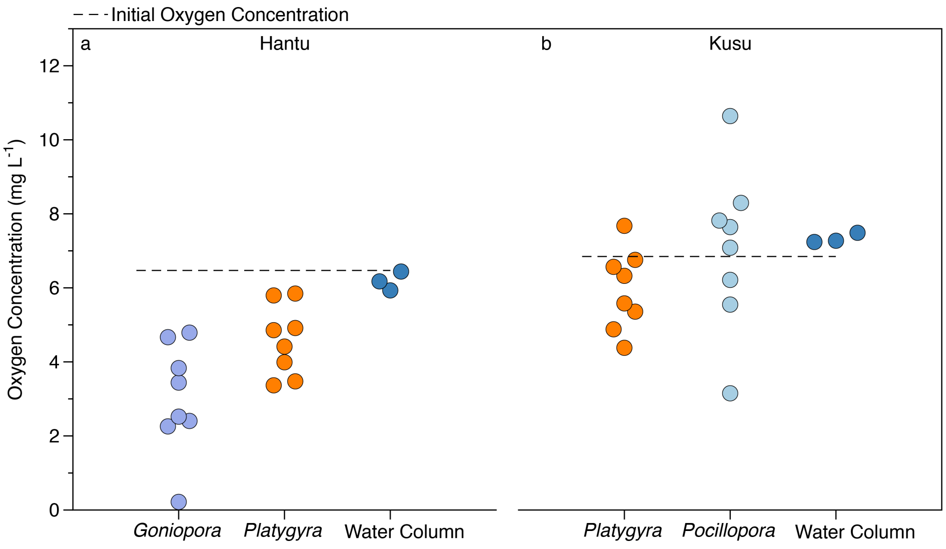


Figure S4: Final oxygen concentrations in each incubation at the end of the experiment at (a) Hantu and (b) Kusu reefs. The dotted line represents the initial oxygen concentration in each incubation.

## 3.5 16S rRNA treemap for DNA- and RNA-based tissue and skeletal communities

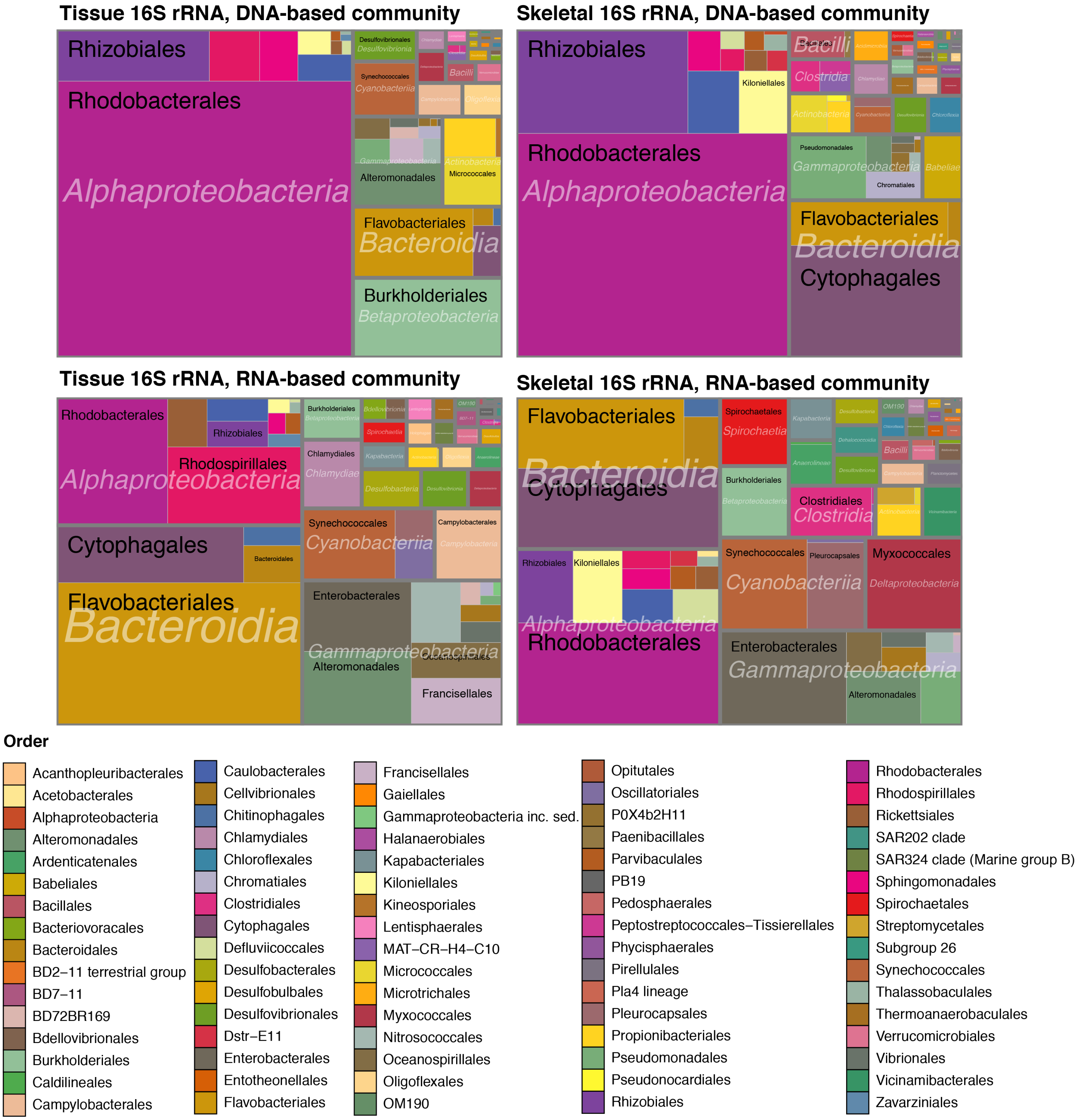


Figure S5: Treemap displaying relative abundance of ASVs grouped by class and order for the 16S rRNA community in DNA-based and RNA-based community samples in coral tissue and skeletal compartments. Rectangles within each treemap are proportional to the relative abundance of each taxonomic order.

## 3.6 16S rRNA relative abundances per sample

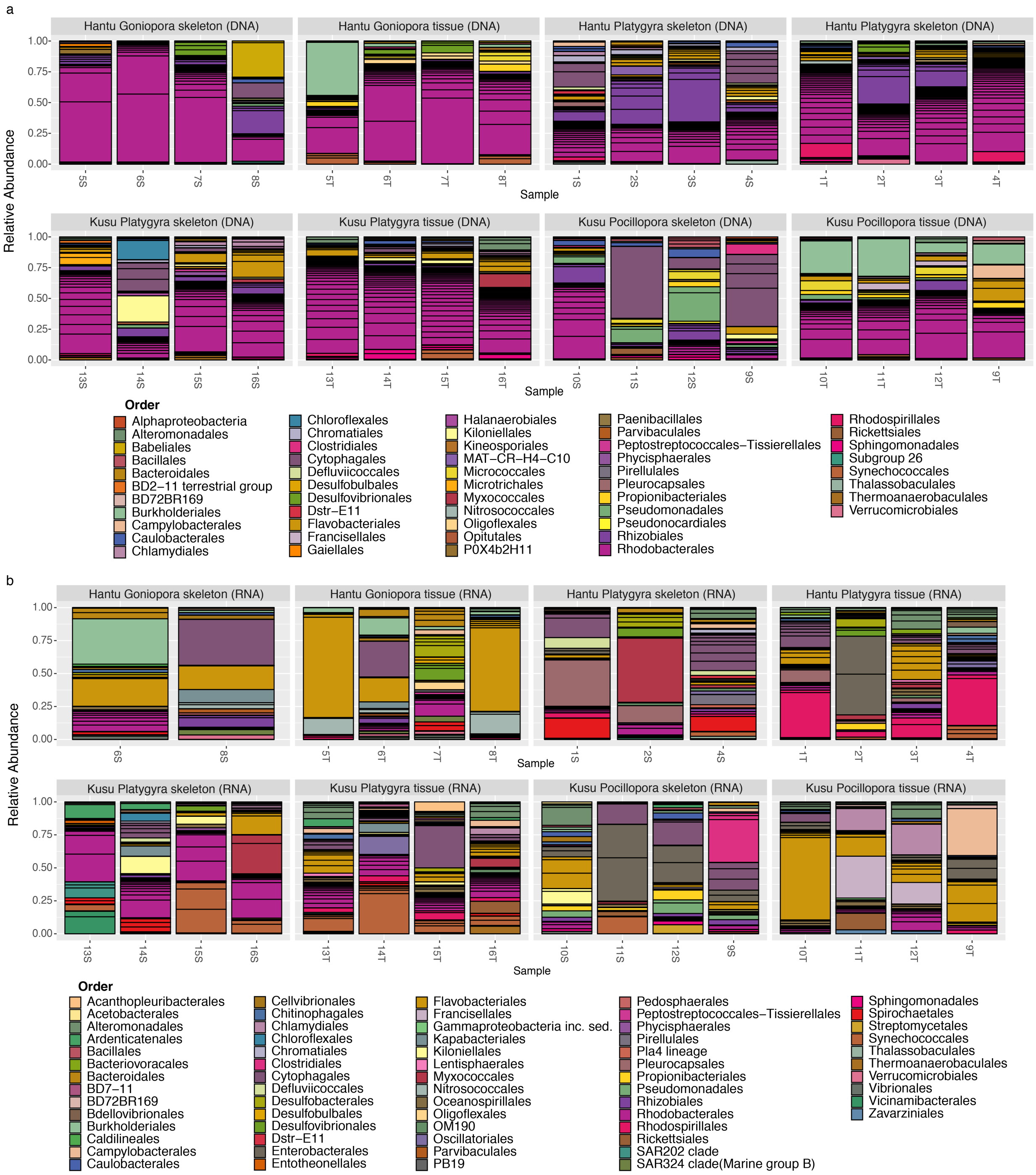


Figure S6: Relative abundance of the 300 most abundant ASVs in 16S rRNA (a) DNA - (b) and RNA - (b) based communities of coral tissue and skeleton samples from individual replicates.

### 3.7 16S rRNA plastid sequences

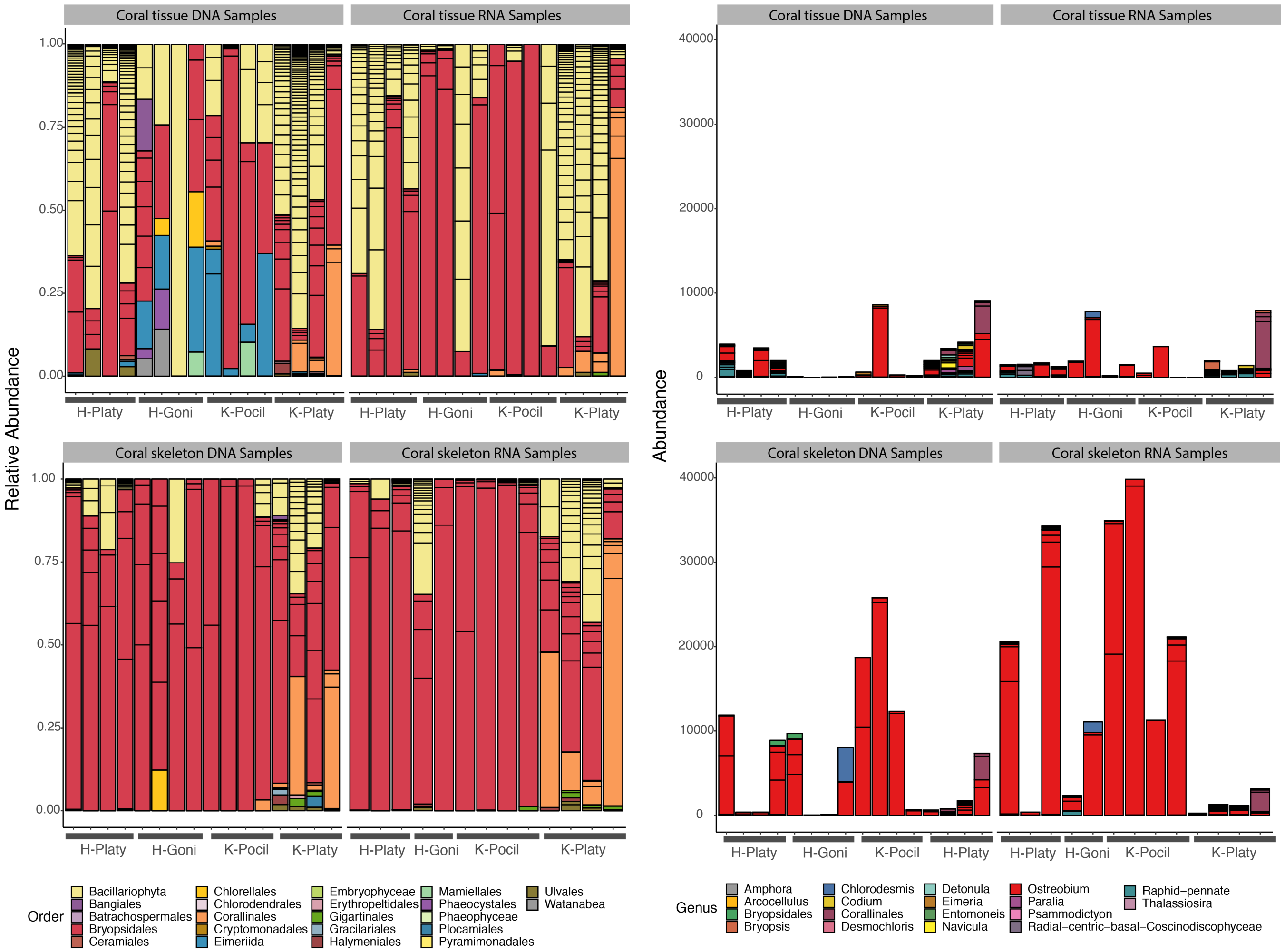


Figure S7: (a) Normalized and (b) raw abundance of 16S rRNA derived plastid sequences for both DNA and RNA samples from coral tissue and skeleton samples. These ASVs were removed from the 16S rRNA community analysis. Only the 50 most abundant taxa are shown at the genus level (b). Samples are labeled by site (H-Hantu or K- Kusu), species (Platy-*Platygyra*; Goni-*Goniopora*; Pocil-*Pocillopora*). Groupings along the x-axis indicate of replicate samples.

## 3.8 Coral tissue 18S rRNA community relative abundance

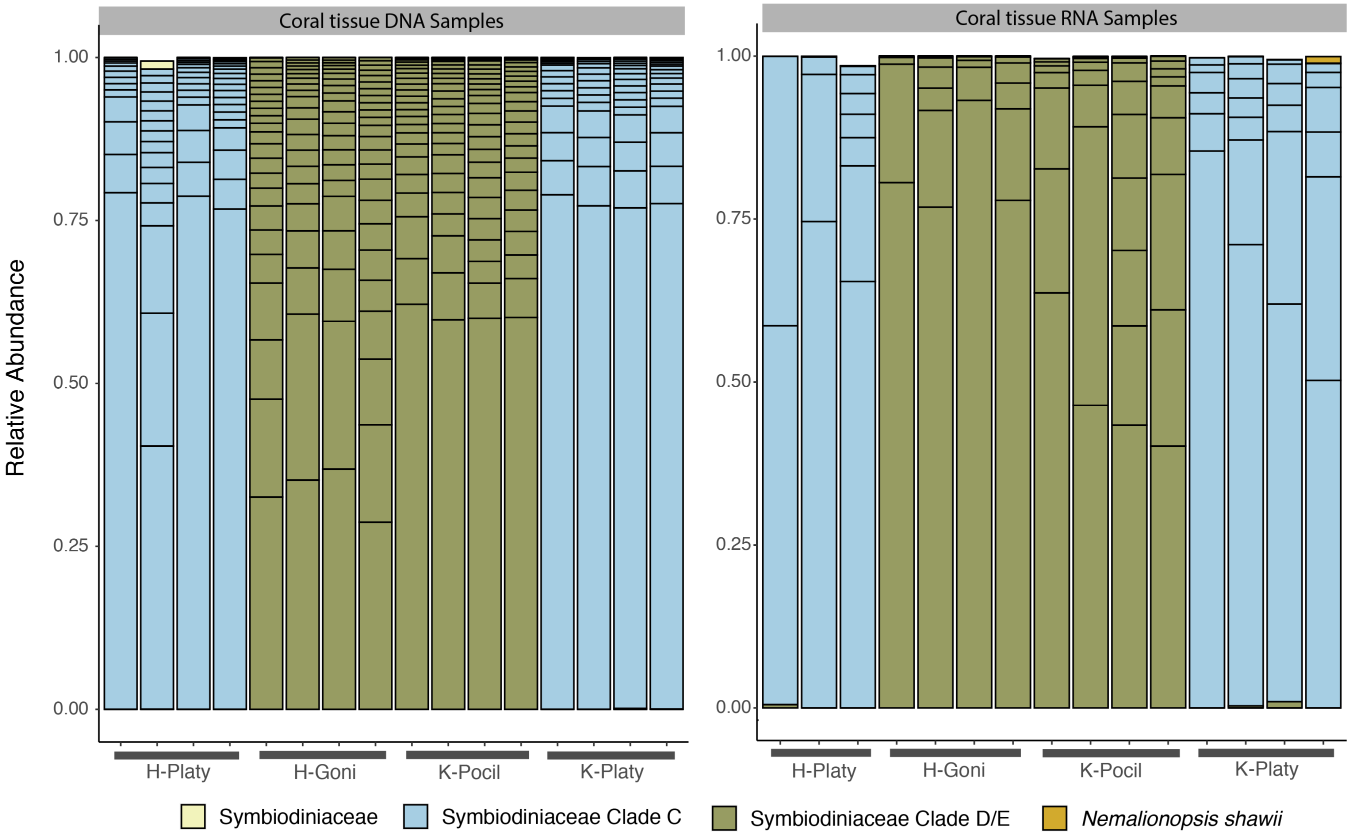


Figure S8: Relative abundance of the 100 most abundant ASVs in 18S rRNA DNA- and RNA- based communities from coral tissue. Samples are labeled by site (H-Hantu or K- Kusu), species (Platy-*Platygyra*; Goni-*Goniopora*; Pocil-*Pocillopora*). Groupings along the x-axis indicate of replicate samples. For the 18S rRNA region sequenced, Clade D/E cannot be distinguished.

### 3.9 18S rRNA PCoA by species and type

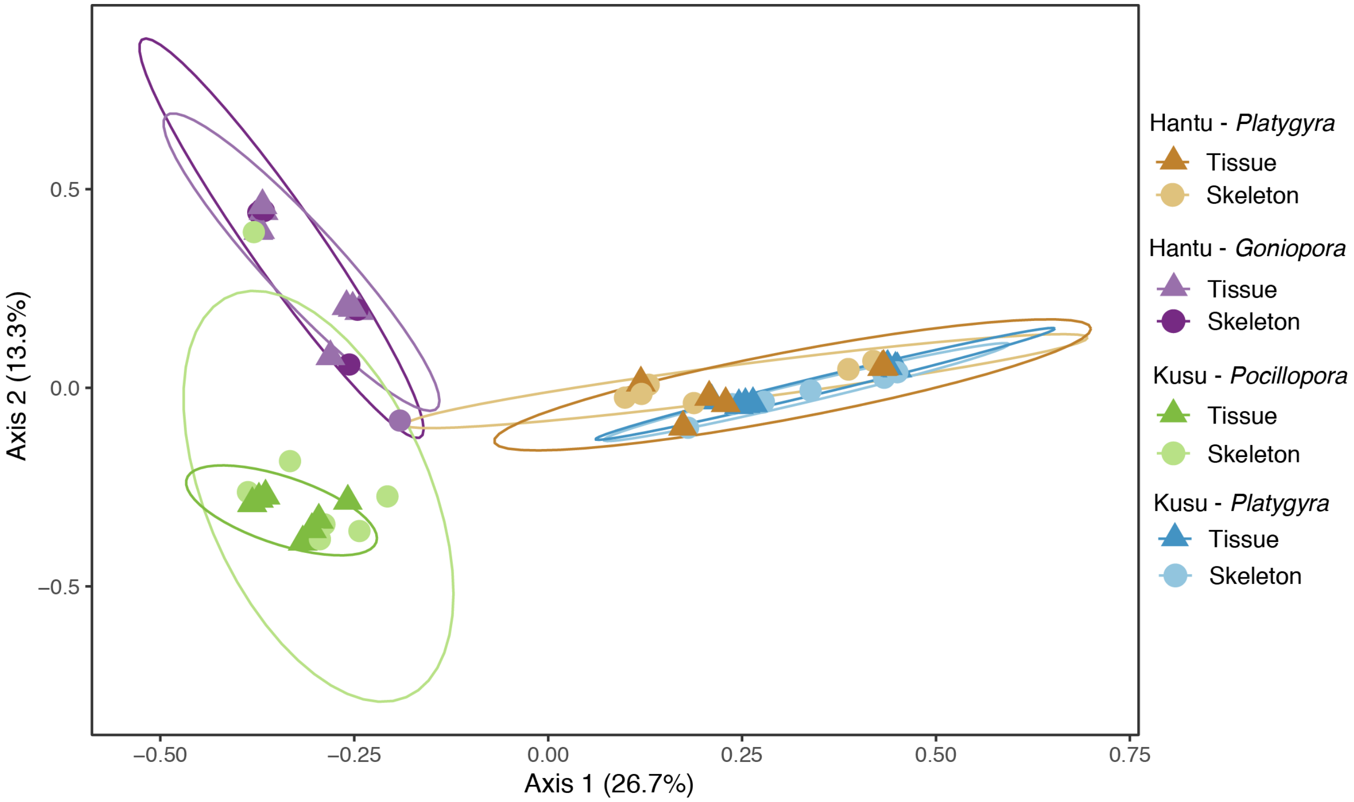


Figure S9: Principal Coordinates Analysis (PCoA) of the 18S rRNA community from coral skeleton and tissue RNA and DNA samples for each species from each site.

## 3.10 18S rRNA non-Symbiodiniaceae treemaps for DNA- and RNA-based tissue and skeletal communities

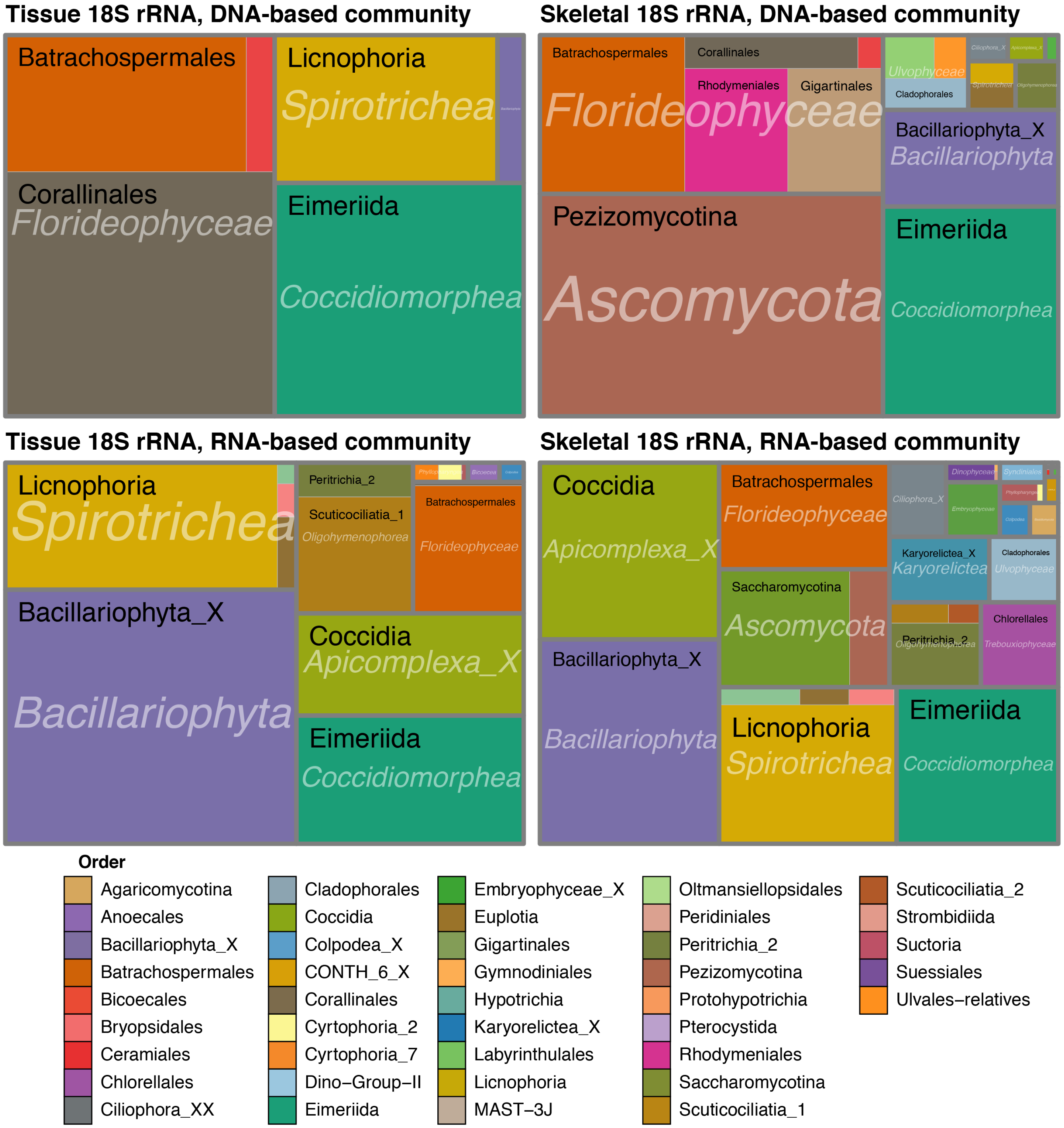


Figure S10: Treemap displaying relative abundance of non-Symbiodiniaceae 18S rRNA ASVs grouped by class and order in DNA-based and RNA-based community samples in coral tissue and skeletal compartments. Rectangles within each treemap are proportional to the relative abundance of each taxonomic order.

## 3.11 16S rRNA seawater community treemap

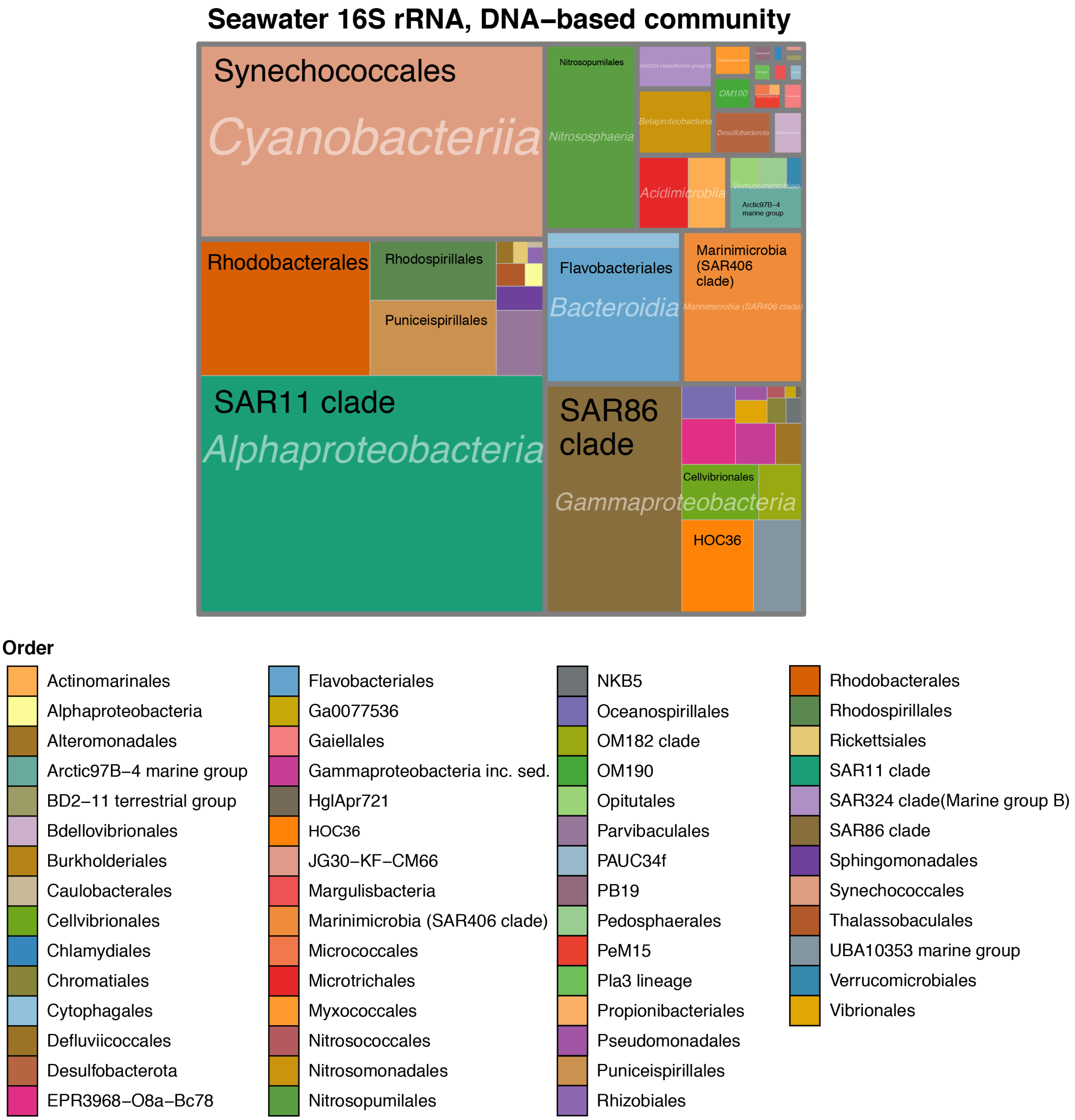


Figure S11: Treemap displaying relative abundance of ASVs grouped by taxonomic class and order for the 16S rRNA community in DNA-based seawater samples. Rectangles within each treemap are proportional to the relative abundance of each taxonomic order.

### 3.12 18S and 16S rRNA plastid seawater community treemaps

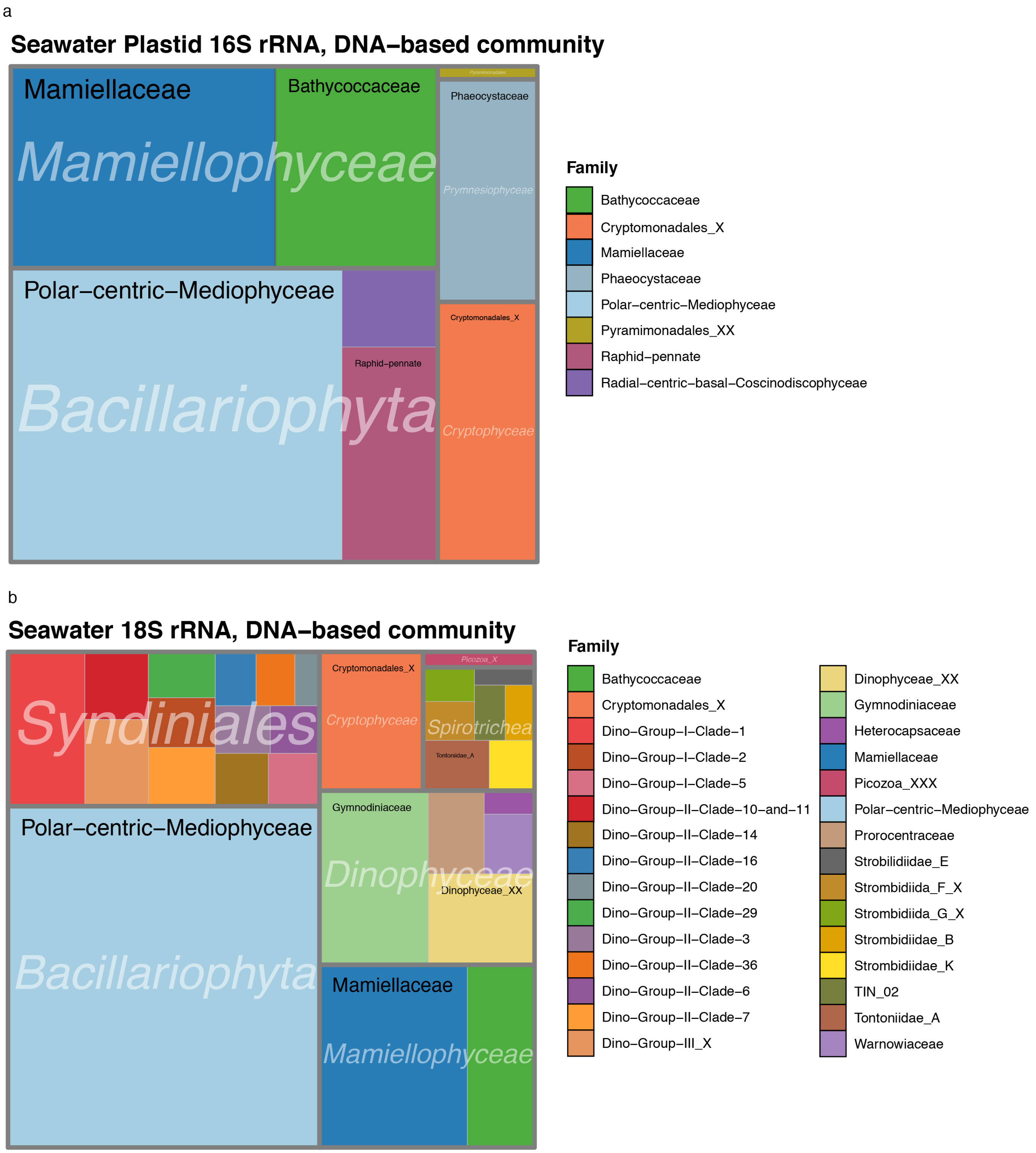


Figure S12: Treemap displaying relative abundance of (a) 18S rRNA and (b) 16S rRNA plastid ASVs grouped by taxonomic order and family in DNA-based seawater samples. Rectangles within each treemap are proportional to the relative abundance of each taxonomic family.

### 3.13 *nifH* Cluster abundance

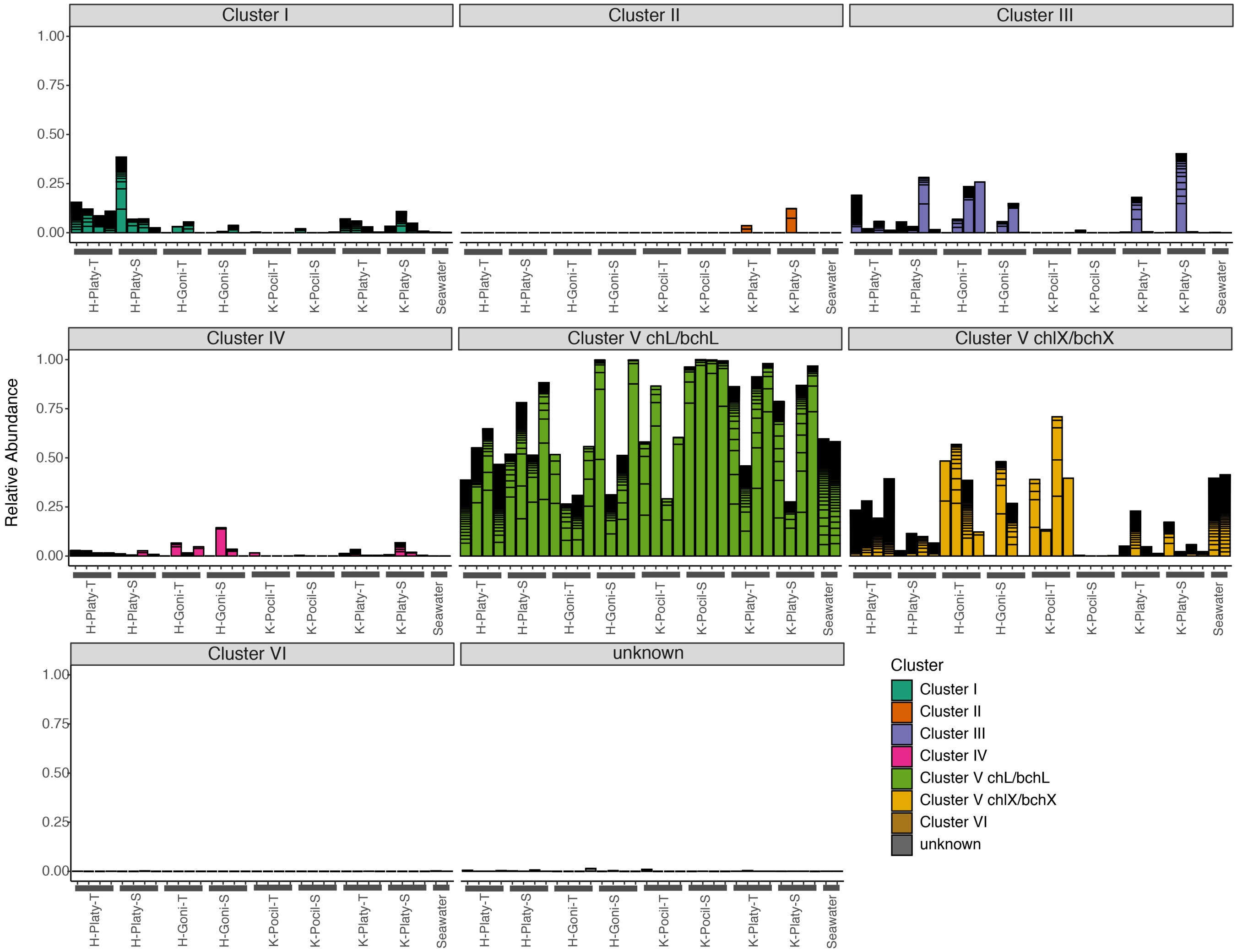


Figure S13: Bar plot of *nifH* Cluster relative abundances for each sample from the total community (DNA). Samples are labeled by site (H-Hantu or K- Kusu), species (Platy-*Platygyra*; Goni-*Goniopora*; Pocil-*Pocillopora*), and type (T-tissue or S-skeleton). Groupings along the x-axis indicate of replicate samples.

## 3.14 *nifH* RNA-based Clusters I–III

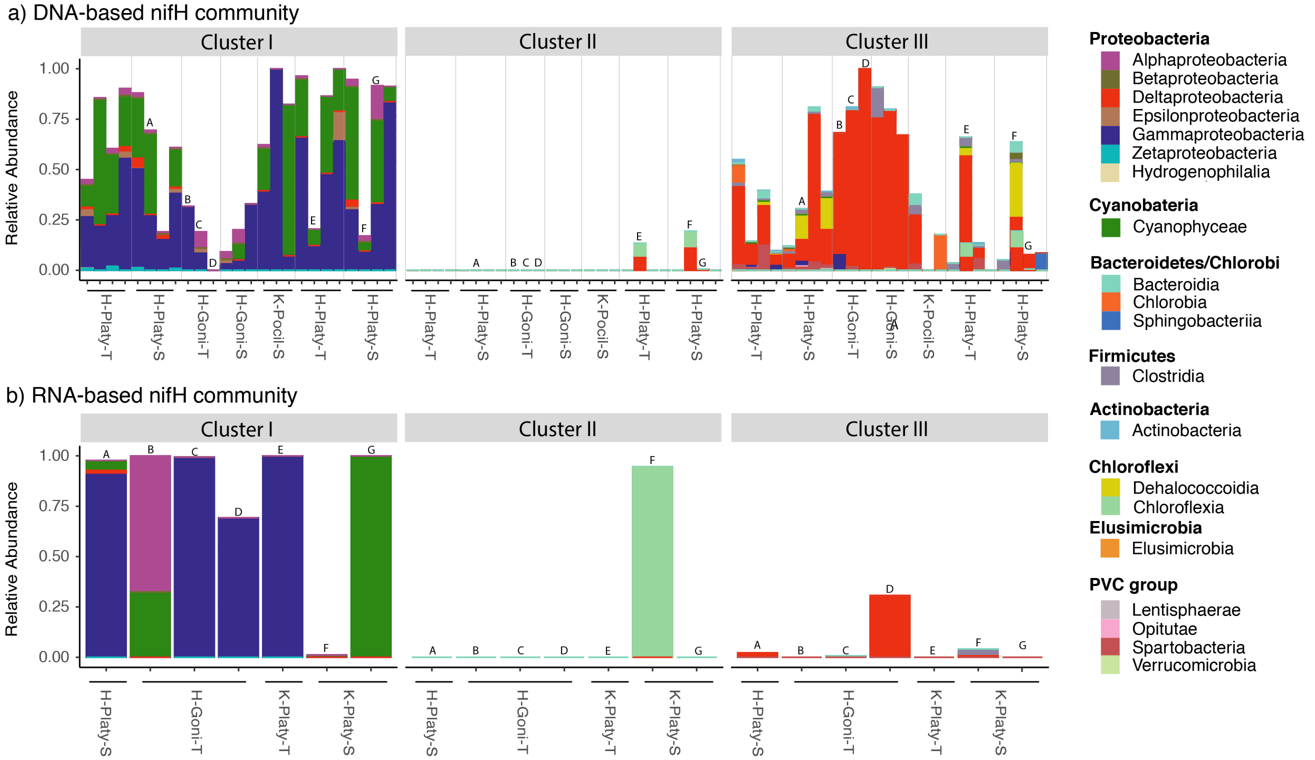


Figure S14: Cluster I – III ASVs from DNA-based and RNA-based *nifH* community samples. Letters represent paired samples between DNA-based and RNA-based communities. Samples are labeled by site (H-Hantu or K- Kusu), species (Platy-*Platygyra*; Goni-*Goniopora*; Pocil-*Pocillopora*), and type (T-tissue or S-skeleton). Groupings along the x-axis indicate of replicate samples

### 3.15 Select RNA:DNA ratios of coral 16S rRNA community

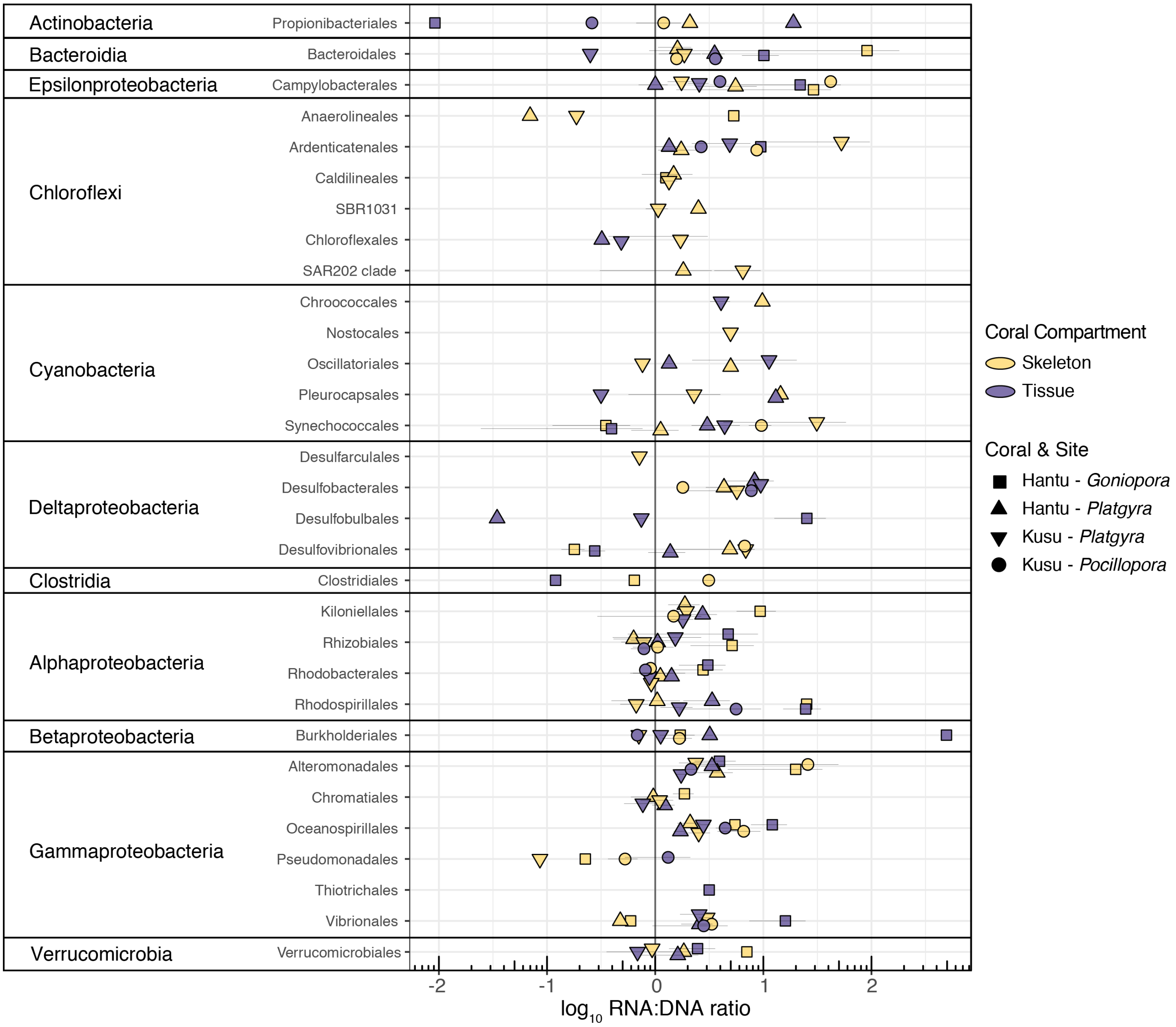


Figure S15: $\text{Log}_{10}$ of 16S rRNA-derived RNA:DNA ratios (16S RNA:DNA) for select taxonomic groups from coral tissue and skeletal samples. Error bars represent the standard error of 16S RNA:DNA ratios for individual ASVs within the same taxonomic order. A ratio of 1 is indicated by the black line.

### 3.16 RNA:DNA ratios of coral *nifH* community

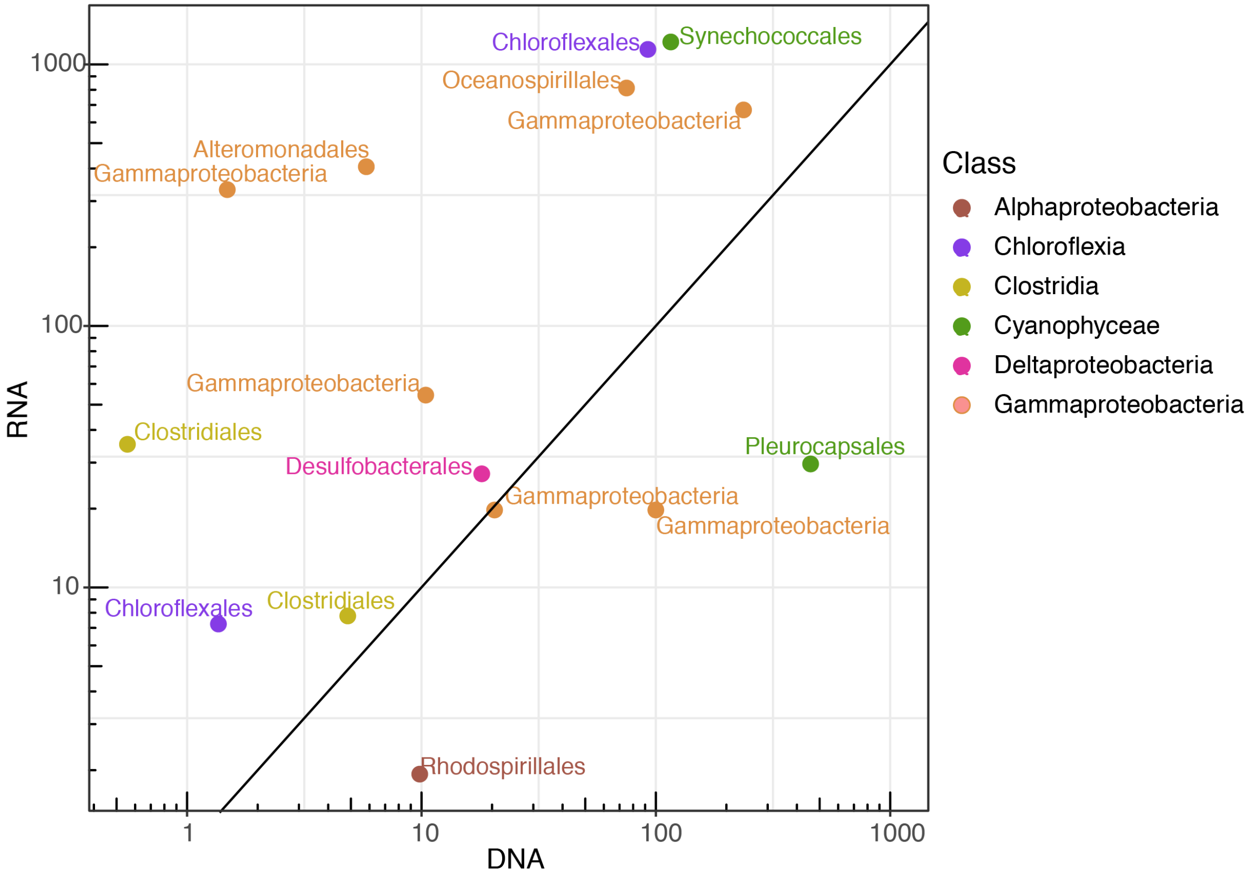


Figure S16: Cluster I - III *nifH* RNA:DNA ratios from ASVs of paired DNA- and RNA- *nifH* samples (n = 7). Samples included are Hantu *Platygyra* skeleton, Hantu *Goniopora* tissue, and Kusu *Platygyra* tissue and skeleton. A *nifH* RNA:DNA ratio of 1 is indicated by the black line.

## References


[1] Mohr W, Großkopf T, Wallace DWR, LaRoche J. Methodological underestimation of oceanic nitrogen fixation rates. PLoS ONE. 2010;5:e12583.

[2] Klawonn I, Lavik G, Böning P, Marchant HK, Dekaezemacker J, Mohr W, et al. Simple approach for the preparation of $^{15-15}N_2$-enriched water for nitrogen fixation assessments: evaluation, application and recommendations. Front microbiol. 2015;6:769.

[3] Bednarz VN, Grover R, Maguer JF, Fine M, Ferrier-Pagès C. The assimilation of diazotroph-derived nitrogen by scleractinian corals depends on their betabolic status. MBio. 2017;8.

[4] Bombar D, Paerl RW, Anderson R, Riemann L. Filtration via conventional glass fiber filters in $^{15}N_2$ tracer assays fails to capture all nitrogen-fixing prokaryotes. Front Mar Sci. 2018;5:e00929–11.

[5] Pupier CA, Bednarz VN, Grover R, Fine M, Maguer JF, Ferrier-Pagès C. Divergent capacity of scleractinian and soft corals to assimilate and transfer diazotrophically derived nitrogen to the reef environment. Front microbiol. 2019;10:1860.

[6] Montoya JP, Voss M, Kahler P, Capone DG. A simple, high-precision, high-sensitivity tracer assay for $N_2$ fixation. Appl Environ Microbiol. 1996;62:986–993.

[7] Januchowski-Hartley FA, Bauman AG, Morgan KM, Seah JCL, Huang D, Todd PA. Accreting coral reefs in a highly urbanized environment. Coral Reefs. 2020;39:717–731.

[8] Sunagawa S, Woodley CM, Medina M. Threatened corals provide underexplored microbial habitats. PLoS ONE. 2010;5:e9554.

[9] Jacobs J, Rhodes M, Sturgis B, Wood B. Influence of environmental gradients on the abundance and distribution of *Mycobacterium* spp. in a Coastal Lagoon Estuary. Appl Environ Microbiol. 2009;75:7378–7384.

[10] Comeau AM, Li WKW, Tremblay JÉ, Carmack EC, Lovejoy C. Arctic ocean microbial community structure before and after the 2007 record sea ice minimum. PLoS ONE. 2011;6:e27492.

[11] Kwong WK, del Campo J, Mathur V, Vermeij MJA, Keeling PJ. A widespread coral-infecting apicomplexan with chlorophyll biosynthesis genes. Nature. 2019;568:103–107.

[12] Weiler BA. Bacterial Communities in tissues and surficial mucus of the cold-water coral *Paragorgia arborea*. Front Mar Sci. 2018;5:378.

[13] Comeau AM, Douglas GM, Langille MGI. Microbiome helper: a custom and streamlined workflow for microbiome research. mSystems. 2017;2.

[14] Gaby JC, Buckley DH. A comprehensive evaluation of PCR primers to amplify the *nifH* gene of nitrogenase. PLoS ONE. 2012;7:e42149.

[15] Bower SM, Carnegie RB, Goh B, Jones SRM, Lowe GJ, Mak MWS. Preferential PCR amplification of parasitic protistan small subunit rDNA from metazoan tissues. J Eukaryot Microbiol. 2004;51:325–332.

[16] Piredda R, Tomasino MP, D'Erchia AM, Manzari C, Pesole G, Montresor M, et al. Diversity and temporal patterns of planktonic protist assemblages at a Mediterranean long term ecological research site. FEMS Microbiol Ecol. 2016;93:fiw200.

[17] Callahan BJ, McMurdie PJ, Rosen MJ, Han AW, Johnson AJA, Holmes SP. DADA2: High-resolution sample inference from Illumina amplicon data. Nat Methods. 2016;13:581–583.

[18] Quast C, Pruesse E, Yilmaz P, Gerken J, Schweer T, Yarza P, et al. The SILVA ribosomal RNA gene database project: improved data processing and web-based tools. Nucleic Acids Res. 2012;41:D590–D596.

[19] McLaren, MR. Silva SSU taxonomic training data formatted for DADA2 (Silva version 138). Zenodo; 2020.

[20] Guillou L, Bachar D, Audic S, Bass D, Berney C, Bittner L, et al. The protist ribosomal reference database (PR2): a catalog of unicellular eukaryote small sub-unit rRNA sequences with curated taxonomy. Nucleic Acids Res. 2013;41:D597–D604.

[21] Moynihan, MA. moyn413/nifHdada2: *nifH* dada2 reference database, v1.1.0. Zenodo; 2020. Available from: https://doi.org/10.5281/zenodo.3964214.

[22] Davis NM, Proctor DM, Holmes SP, Relman DA, Callahan BJ. Simple statistical identification and removal of contaminant sequences in marker-gene and metagenomics data. Microbiome. 2018;6:1–14.

[23] Raymond J, Siefert JL, Staples CR, Blankenship RE. The natural history of nitrogen fixation. Mol Biol Evol. 2004;21:541–554.

[24] Méheust R, Castelle CJ, Carnevali PBM, Farag IF, He C, Chen LX, et al. Groundwater *Elusimicrobia* are metabolically diverse compared to gut microbiome *Elusimicrobia* and some have a novel nitrogenase paralog. ISME J. 2020;14:2907–2922.

[25] Heller P, Tripp JH, Turk-Kubo K, Zehr JP. ARBitrator: a software pipeline for on-demand retrieval of auto-curated *nifH* sequences from GenBank. Bioinformatics. 2014;30:2883–2890.

[26] Pogoreutz C, Rädecker N, Cárdenas A, Gärdes A, Voolstra CR, Wild C. Sugar enrichment provides evidence for a role of nitrogen fixation in coral bleaching. Glob Chang Biol. 2017;23:3838–3848.

[27] Shashar N, Cohen Y, Loya Y, Sar N. Nitrogen fixation (acetylene reduction) in stony corals: evidence for coral-bacteria interactions. Mar Ecol Prog Ser. 1994;111:259–264.

[28] Lesser MP, Falcón LI, Rodríguez-Román A, Enríquez S, Hoegh-Guldberg O, Iglesias-Prieto R. Nitrogen fixation by symbiotic cyanobacteria provides a source of nitrogen for the scleractinian coral *Montastraea cavernosa*. Mar Ecol Prog Ser. 2007;346:143–152.

[29] Davey M, Holmes G, Johnstone R. High rates of nitrogen fixation (acetylene reduction) on coral skeletons following bleaching mortality. Coral Reefs. 2007;27:227–236.

[30] Grover R, Ferrier-Pagès C, Maguer JF, Ezzat L, Fine M. Nitrogen fixation in the mucus of Red Sea corals. J Exp Biol. 2014;217:3962–3963.

[31] Bednarz VN, Cardini U, van Hoytema N, Al-Rshaidat M, Wild C. Seasonal variation in dinitrogen fixation and oxygen fluxes associated with two dominant zooxanthellate soft corals from the northern Red Sea. Mar Ecol Prog Ser. 2015;519:141–152.

[32] Cardini U, Bednarz V, Naumann MS, van Hoytema N, Rix L, Foster RA, et al. Functional significance of dinitrogen fixation in sustaining coral productivity under oligotrophic conditions. Proc R Soc B. 2015;282:20152257.

[33] Cardini U, Bednarz VN, van Hoytema N, Rovere A, Naumann MS, Al-Rshaidat MMD, et al. Budget of primary production and dinitrogen fixation in a highly seasonal Red Sea coral reef. Ecosystems. 2016;19:771–785.

[34] Cardini U, van Hoytema N, Bednarz VN, Rix L, Foster RA, Al-Rshaidat MMD, et al. Microbial dinitrogen fixation in coral holobionts exposed to thermal stress and bleaching. Environ Microbiol. 2016;18:2620–2633.

[35] Benavides M, Houlbrèque F, Camps M, Lorrain A, Grosso O, Bonnet S. Diazotrophs: a non-negligible source of nitrogen for the tropical coral *Stylophora pistillata*. J Exp Biol. 2016;219:2608–2612.

[36] Pogoreutz C, Rädecker N, Cárdenas A, Gärdes A, Wild C, Voolstra CR. Nitrogen fixation aligns with *nifH* abundance and expression in two coral trophic functional groups. Front microbiol. 2017;8:1187.

[37] Sangsawang L, Casareto BE, Ohba H, Vu HM, Meekaew A, Suzuki T, et al. $^{13}C$ and $^{15}N$ assimilation and organic matter translocation by the endolithic community in the massive coral *Porites lutea*. R Soc Open Sci. 2017;4:171201.

[38] Bednarz VN, Naumann MS, Cardini U, van Hoytema N, Rix L, Al-Rshaidat MMD, et al. Contrasting seasonal responses in dinitrogen fixation between shallow and deep-water colonies of the model coral *Stylophora pistillata* in the northern Red Sea. PLoS ONE. 2018;13:e0199022.

[39] Bednarz VN, van de Water JAJM, Rabouille S, Maguer JF, Grover R, Ferrier-Pagès C. Diazotrophic community and associated dinitrogen fixation within the temperate coral *Oculina patagonica*. Environ Microbiol. 2018;21:480–495.

[40] Lesser MP, Morrow KM, Pankey SM, Noonan SHC. Diazotroph diversity and nitrogen fixation in the coral *Stylophora pistillata* from the Great Barrier Reef. ISME J. 2018;12:813–824.

[41] Tilstra A, El-Khaled YC, Roth F, Rädecker N, Pogoreutz C, Voolstra CR, et al. Denitrification aligns with $N_2$ fixation in Red Sea corals. Sci Rep. 2019;9:1–9.

[42] Meunier V, Bonnet S, Pernice M, Benavides M, Lorrain A, Grosso O, et al. Bleaching forces coral's heterotrophy on diazotrophs and *Synechococcus*. ISME J. 2019;13:2882–2886.

[43] El-Khaled YC, Roth F, Tilstra A, Rädecker N, Karcher DB, Kürten B, et al. In situ eutrophication stimulates dinitrogen fixation, denitrification, and productivity in Red Sea coral reefs. Marine Ecology Progress Series. 2020;645:55–66.

[44] Morgan KM, Moynihan MA, Sanwlani N, Switzer AD. Light limitation and depth-variable sedimentation drives vertical reef compression on turbid coral reefs. Front Mar Sci. 2020;7:571256.

[45] Bauman AG, Hoey AS, Dunshea G, Feary DA, Low J, Todd PA. Macroalgal browsing on a heavily degraded, urbanized equatorial reef system. Sci Rep. 2017;7:1–8.

[46] Huang D, Tun K, Chou L, Todd PA. An inventory of zooxanthellate scleractinian corals in Singapore, including 33 new records. Raffles Bull Zool. 2009;22:69–80.

[47] House JE, Brambilla V, Bidaut LM, Christie AP, Pizarro O, Madin JS, et al. Moving to 3D: relationships between coral planar area, surface area and volume. PeerJ. 2018;6:e4280.
